# Cognitive outcomes following coronary artery bypass grafting: A systematic review and meta-analysis of 91,829 patients

**DOI:** 10.1016/j.ijcard.2019.04.065

**Published:** 2019-08-15

**Authors:** Danielle Greaves, Peter J. Psaltis, Tyler J. Ross, Daniel Davis, Ashleigh E. Smith, Monique S. Boord, Hannah A.D. Keage

**Affiliations:** a Cognitive Ageing and Impairment Neurosciences Laboratory, School of Psychology, Social Work and Social Policy, University of South Australia, Adelaide, Australia; b Vascular Research Centre, Heart Health Theme, South Australian Health and Medical Research Institute, Adelaide, Australia and Adelaide Medical School, University of Adelaide, Adelaide, Australia; c MRC Unit for Lifelong Health and Ageing Unit at University College London, London, United Kingdom; d Alliance for Research in Exercise, Nutrition and Activity, School of Health Sciences, University of South Australia, Adelaide, Australia

**Keywords:** Coronary artery bypass grafting surgery, Cardiac surgery, Post-operative cognitive decline, Delirium, Dementia

## Abstract

**Background:**

Cognitive impairments, including delirium, are common after coronary artery bypass grafting (CABG) surgery, as described in over three decades of research. Our aim was to pool estimates across the literature for the first-time, relative to time (from pre- to post-CABG) and diagnosis (cognitive impairment, delirium and dementia).

**Methods:**

A systematic search of four databases was undertaken. 215 studies incorporating data from 91,829 patients were used to estimate the prevalence of cognitive impairments pre- and post-CABG, including delirium and dementia post-CABG, using random effects meta-analyses.

**Results:**

Pre-surgical cognitive impairment was seen in 19% of patients. Post-operatively, cognitive impairment was seen in around 43% of patients acutely; this resolved to 19% at 4–6 months and then increased to 25% of patients between 6-months to 1-year post-operatively. In the long term, between 1 and 5-years post-operatively, cognitive impairment increased and was seen in nearly 40% of patients. Post-operative delirium was apparent in 18% of CABG patients which increased to 24% when a diagnostic instrument was utilized alongside clinical criteria. Dementia was present in 7% of patients 5–7 years post-surgery.

**Conclusion:**

The results of this meta-analysis demonstrate that cognitive impairment and delirium are major issues in CABG patients which require specific attention. It is imperative that appropriate methods for investigating cognitive impairment, and screening for delirium using a diagnostic instrument, occur in both pre-and post-CABG settings.

## Highlights

• This meta-analysis pooled results of 91,829 patients, including 215 studies.
• Cognitive impairment and delirium are seen in up to 40% of CABG patients.
• Appropriate methods investigating cognitive impairment are crucial post-CABG.
• Appropriate methods of screening for delirium are crucial post-CABG.

## 1 Introduction

Coronary artery bypass grafting (CABG) improves coronary vascularization, myocardial ischemia, cardiac function and cardiac-related mortality rates [1]. Despite these benefits, CABG is also associated with high risks of post-operative cognitive impairment and decline [[2], [3], [4], [5]]. Understanding the extent of cognitive impairment and the likely time course for recovery (or otherwise) is important, but remains unclear.

Cognitive impairments after CABG are associated with difficulties completing activities of daily living [6], higher mortality, early retirement and higher dependency following discharge [7]. Some post-operative cognitive impairment is likely attributable to delirium immediately following CABG [8]. Delirium is characterized as an acute and fluctuating deficit in attention and arousal [9]. Although an acute syndrome, delirium is associated with long-term impairments in overall function, cognitive function and quality of life, along with increased mortality, hospital stay and readmissions [[9], [10], [11], [12]].

Understanding the extent of cognitive impairments in the context of CABG, including delirium, and the likely time course for recovery (or otherwise) is important, but remains unclear. Previous meta-analyses have been overly restrictive, employing limitations on publication date and neuropsychological tests [13], or only comparing between cardiopulmonary bypass to no bypass [14]. Although these studies have provided information regarding predictors of cognitive deficits, these search restrictions have limited the generalisability of results. No meta-analysis has investigated delirium or dementia following CABG. Moreover, when reporting cognitive change, various metrics have been used: (i) 1 standard deviation method (1SD) [15], (ii) 20% decline method (20:20) [16], (iii) reliable change index methods (RCI) [[17], [18], [19], [20]] and (iv) specific cut-offs. Though there is no consensus on which is most clinically relevant, considering the approach to measuring cognitive outcomes is an important factor when comparing studies. This review will include all studies on cognitive impairment (including delirium and dementia) in CABG patients (pre- and post-operatively), which encompass a wide range of assessment approaches and time points, to provide a complete picture of cognitive impairments in the context of CABG.

This systematic review and meta-analysis aims to determine the prevalence of pre and post-operative cognitive outcomes, including delirium and dementia, across time, from pre-operatively to 5 years and beyond post-operatively. We also aimed to quantify the effect of classification methods for cognitive outcomes (1SD, 20:20, RCI and cut-off) and delirium (clinical criteria with and without a standardized instrument) on prevalence estimates. Describing the time course of post-operative cognitive outcomes has broad clinical implications, e.g. consent, mortality, quality of life and dependency.

## 2 Method

### 2.1 Search strategy

We followed the Preferred Reporting Items for Systematic Reviews and Meta-Analysis (PRISMA) guidelines [21]. Article selection and data extraction were undertaken by at least two reviewers (between DG, MSB, TJR), with disagreements resolved by consensus.

We searched Medline, PsycINFO, EMBASE and the Cochrane databases (12th March 2017) using the Ovid platform when possible. Search terms and medical subject headings (used when possible) utilized were: Coronary Artery Bypass/ OR “coronary artery bypass” OR CABG AND Cognition/ OR Delirium/ OR Dementia/ OR Alzheimer Disease/ OR Neuropsychological Tests/ OR Cognit* OR Deliri* OR Dementia* OR Alzheimer* OR MCI or mild cognitive impairment* OR mild-cognitive impairment* OR neuropsycholo* OR POCD OR postoperative cognitive OR post-operative cognitive OR MMSE OR mini-mental state examination OR cerebral function OR neurocognit* OR encephalopath*.

### 2.2 Study eligibility

Inclusion criteria were: peer-reviewed, full-text, English language studies which investigated patients undergoing CABG surgery or reported results of those who had undergone CABG surgery. Studies also needed to report a cognitive outcome (either by a standardized test result, or neuropsychological battery, or by a clinical diagnosis of: delirium, mild cognitive impairment, dementia or Alzheimer's disease).

Exclusion criteria included: case series (*n* < 5), dissertations, book chapters, protocol papers, reviews, news articles, conference abstracts, letters to the editor, editorials and comment publications; no description of their operationalization (or definition used for cognitive decline), or incomplete reporting in respect to pre- and post-operative cognitive outcomes. Additionally, if multiple studies investigated the same cohort duplicate samples were excluded.

### 2.3 Quality assessment

We combined two existing checklists used for assessing prevalence data studies (Joanna Briggs Institute) [22,23]. Higher scores indicated greater overall study design and reporting quality (range 0–14).

### 2.4 Data extraction

Data extracted included: country, sample size, age, gender, cognitive impairment/delirium/dementia assessment criteria, frequency of cognitive impairment or decline/delirium/dementia relative to time periods (1–7 days post-surgery for delirium, for cognition both pre-surgery and post-surgery: immediate post-surgery to 4-days, 5-days to 1-month post-surgery, 1-month to 4-months post-surgery, 4-months to 6-months post-surgery, 6-months to 1-year post-surgery, 1-year to 3-years post-surgery, 3-years to 5-years post-surgery and >5 years post-surgery).

### 2.5 Statistical analyses

Demographic data were calculated from the reported pre-operative samples. When age was reported separately for multiple groups in a single study, the pooled age mean and standard deviation (SD) for each study was calculated [24]. The I^2^ statistic was used to measure study heterogeneity and classified as low (I^2^ = 25%–50%), moderate (I^2^ = 50%–75%) or high (I^2^ ≥ 75%) using classification criteria suggested by Higgins et al. [25]. Because of clinical and statistical heterogeneity, we accounted for random effects in our pooled estimates. Publication bias was investigated using funnel plots for main analyses (Supplementary Fig. 1), revealing some small biases within some of the cognition analyses. However, these were not large enough to influence any conclusions drawn.

Separate meta-analyses to estimate prevalence were conducted for cognitive impairment or decline, delirium and dementia. For cognitive impairment, multiple meta-analyses were conducted for differing time-points (pre-surgery, immediate post-surgery to 4-days, 5-days to 1-month post-surgery, 1-month to 4-months post-surgery, 4-months to 6-months post-surgery, 6-months to 1-year post-surgery, 1-year to 3-years, 3-years to 5-years and >5-years post-surgery). Multiple time-points were selected as outcomes rather than a singular time-point. This approach enabled us to capture the variation of cognitive impairments across time. Different time points, from pre- to post-CABG, provide clinicians with a variety of information in the context of consent, acute-care and prognosis. Only one time-point was reported for both delirium and dementia data, where delirium was 1–7 days post-surgery and dementia was not restricted in a time period (all dementia studies included in same analysis). We also estimated the effects of diagnostic approach (delirium) and method of classification (cognitive impairment or decline). Delirium operationalisation was considered as; (i) studies utilising a standardized instrument e.g. Confusion Assessment Method (CAM) or the Delirium Rating Scale (DRS) to inform the reference standard, and (ii) studies not utilising a specific instrument. Cognitive impairment estimates were grouped according to the classification used: (i) 1 standard deviation method (1 SD) [15], (ii) 20% decline method (20:20) [16], (iii) the reliable change index methods (RCI) [[17], [18], [19], [20]], and (iv) cut-off method (Table 1). Any data which did not fall into these categories were used only in the total pooled estimates. All meta-analyses were conducted using Comprehensive Meta-Analysis software (version 3) [28].

**Table 1:** Cognitive impairment/decline definitions utilized for meta-analysis.

Method	Definition utilized in meta-analysis	Sub-definition	Method reference
1 SD	≥1 SD decline in a participant's postoperative test score compared to their preoperative test score, on at least 20% of the tests. The SD is either calculated based on published or sample (pre-operative) norms Method reflects individual change relative to sample or population data (utilized to calculate the SD).		Newman et al. [15]
20:20	≥20% decline in a participant's post-operative test score compared to their preoperative test score, on at least 20% of the tests conducted. Method reflects individual change relative to self.		Stump [16]
RCI⁎	RCI decline of ≥1.64 in ≥20% of tests, or global decline of ≥1.64 in RCI composite score. All versions of RCI calculation methods were included in the analysis. Method reflects individual change relative to sample or population data taking practice effects into account.	Change in participant's preoperative to postoperative test score, divided by the standard error of the difference between the two test scores (SE_difference_ = √ 2((SD_baseline control_√(1 − r_xx_))^2^, where r_xx_ is the test–retest reliability of the measure.	Jacobson and Truax [17]
		Average change in preoperative to postoperative test scores of the control group is subtracted from within-participant change in preoperative to postoperative test scores. This value is then divided by the standard deviation of the control group change.	Rasmussen et al. [18]
		Average change in preoperative to postoperative test scores of the control group is subtracted from within-participant change in preoperative to postoperative test scores. This value is then divided by the standard error of the difference between the test scores (SE_difference_ = √2((SD_baseline control_√(1 − r_xx_))^2^, where r_xx_ is the test–retest reliability of the measure.	Chelune et al. [19]
		Average change in preoperative to postoperative test scores of the control group is subtracted from within-participant change in preoperative to postoperative test scores. This value is then divided by the within-participant standard deviation of the matched control group.	Mollica et al. [20]
Cut-off	Use population norms or a threshold of decline (e.g. decrease by 2 points) to define cognitive impairment in particular tests (e.g., MMSE) Method reflects the individual's performance on a test.		Chakravarthy et al. [26], Goto et al. [27]

Where: 1 SD — 1 standard deviation method, 20:20–20% decline method, RCI — reliable change index method, SD — standard deviation.

⁎ Both 90% (RCI = 1.64) and 95% (RCI = 1.96) intervals are used across studies to define cognitive impairment. Participants that decline by >1.64 or >1.96 (depending on the definition used) on ≥20% of tests or a composite RCI score that aggregates all test scores.

## 3 Results

**Fig. 1:**
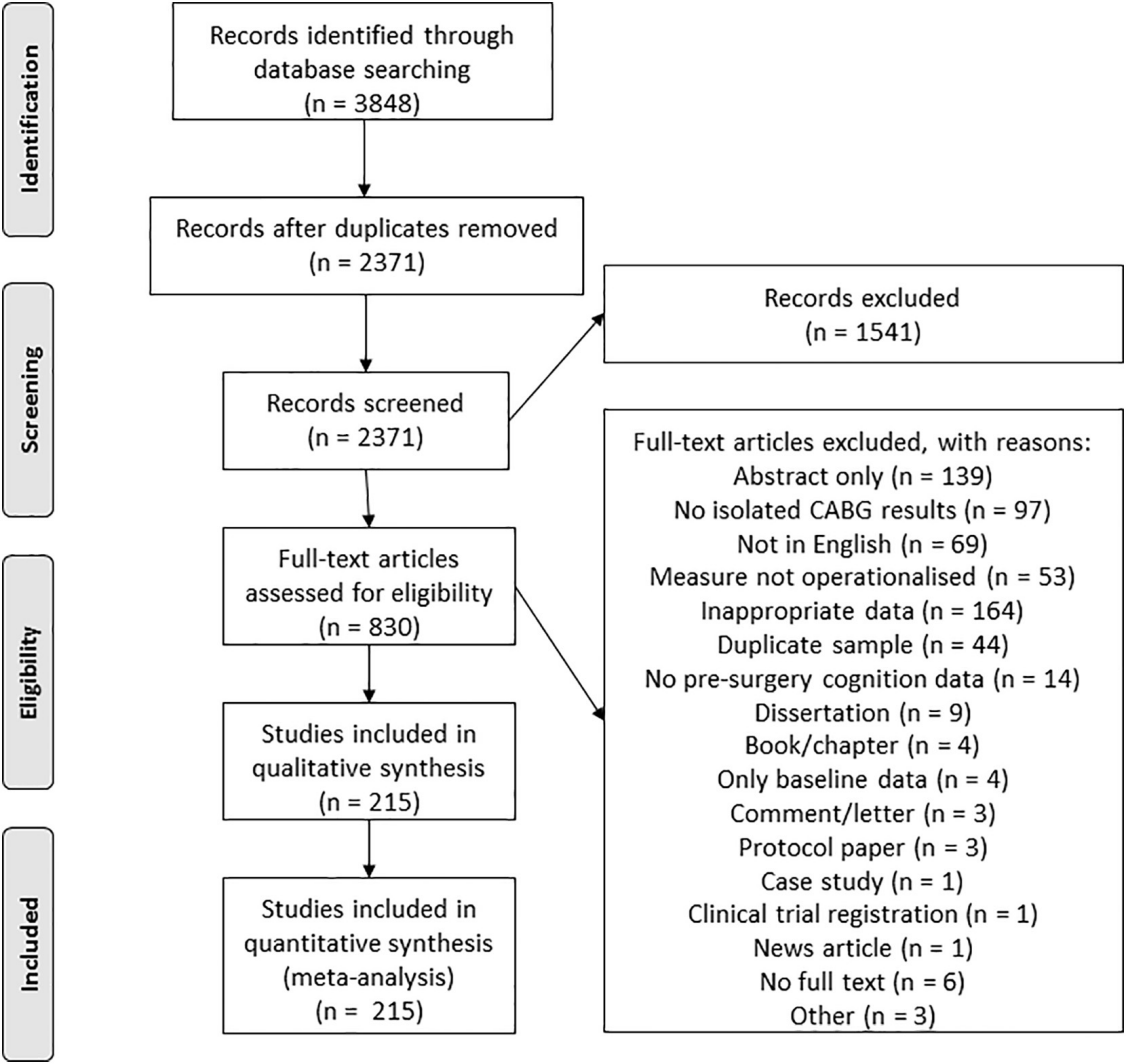
PRISMA flow diagram.

We identified 3848 articles, 2371 after removing duplicates. A total of 830 papers met initial criteria for full-text review and of these, 215 were included (Fig. 1).

Of the 215 included studies, 39 were conducted in the United States, 20 in the United Kingdom, 20 in Canada, 19 in Japan, 17 in Australia, 14 in Germany and 10 in the Netherlands. The remaining 76 studies were conducted across another 23 countries, including some low-income countries. The earliest study was published in 1983 with the most recent in 2017. There were 6, 20 and 110 studies published in the 1980s, 1990s, and 2000s respectively and 79 studies published from 2010 onwards. Sample sizes ranged from 8 to 14,262 with a mean sample size of 449. The included papers comprised data from 96,903 patients, where 91,829 patients underwent isolated CABG. The mean age across studies was 64.86 years, with 77.84% of patients being male. Overall the included studies were of a good quality according to the critical appraisal assessment conducted, where the average score was 10 (of 14) with a SD of 2, ranging from 4 to 14 (see Supplementary Table 1 for individual study information); no studies were excluded on the basis of quality.

### 3.1 Cognitive impairment and decline

Data from 156 studies investigating cognitive impairment were included in the meta-analysis. Medium to high heterogeneity was present across all analyses (I^2^ range 62.61–97.93) (Supplementary Table 2).

**Fig. 2:**
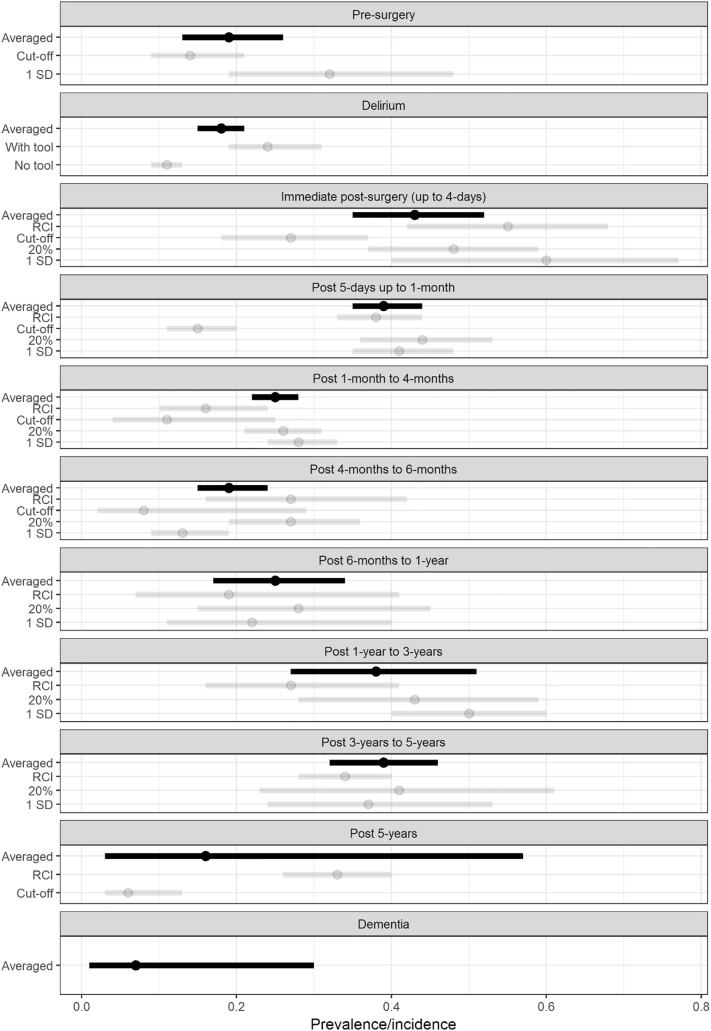
Forest plot of prevalence/incidence relative to outcome (dementia, delirium and cognitive impairment), classification/diagnostic method and time point. Legend: Averaged = total pooled estimate of all studies within the time-point for cognitive outcome (delirium, dementia), RCI = contains reliable change index studies, Cut-off = contains cut-off method studies, 20% = contains 20% method studies, 1SD = contains 1 standard deviation method studies, With tool = utilized standardized diagnostic tools e.g. confusion assessment method, No tool = did not utilize a diagnostic tool.

At each time point, the prevalence of cognitive impairment was: 19% (95%CI 13–26%) pre-operatively (13 studies, *n* = 2274), and 43% (95%CI 35–52%) up to 4-days post-operatively (19 studies, *n* = 1542), 39% (95%CI 35–44%) up to 1-month (88 studies, *n* = 11065), 25% (95%CI 22–28%) up to 4-months (71 studies, *n* = 9658), 19% (95%CI 15–24%) up to 6-months (25 studies, *n* = 3967), 25% (95%CI 17–34%) up to 1-year (11 studies, *n* = 2939), 38% (95%CI 27–51%) between 1 and 3-years (4 studies, *n* = 203), 39% (95%CI 32–46%) 3–5-years (5 studies, *n* = 649) and 16% (95%CI 3–57%) over 5-years post-operatively (2 studies, *n* = 285) (Fig. 2).

Studies classifying cognitive impairment using 1SD or 20:20 methods had higher prevalence estimates and larger heterogeneity than the RCI and cut-off methods, particularly for estimates within the first month following surgery (Fig. 2, Supplementary Table 2–4).

### 3.2 Delirium

Delirium was ascertained in 70 studies (*n* = 61116), with an estimated pooled period prevalence of 18% (95%CI 15–21%) up to one week post-operatively. However, estimates from studies using a standardized instrument to detect delirium were substantially higher with 24% (95% CI 19–31%) compared with 11% (95% CI 9–13%), see Fig. 2.

### 3.3 Dementia

Three studies including 6457 patients reported dementia presence data following CABG surgery. The pooled estimate was 7% (95% CI 1–30%) over 4.9–7.5 years post-CABG surgery, though there was high heterogeneity between studies (I^2^ = 99.17), Supplementary Table 2.

## 4 Discussion

This meta-analysis comprehensively quantifies cognitive impairment in CABG patients, including delirium and dementia, and its variation over follow-up time. Pre-operatively, 19% of patients had a cognitive impairment; this increased to nearly half acutely post-surgery, and then decreased to around a fifth of patients up to a year post-surgery. At 5-years post-surgery, the point prevalence of cognitive impairment is nearly 40%. Though these pooled estimates are not directly comparable across time because they represent different individuals, and therefore should be interpreted with some caution, together they represent a substantial burden of cognitive impairment in the short and long-term associated with CABG. We also found that delirium was a significant part of the cognitive impairment observed, though reports not using a standardized instrument were likely to be under-estimated. Taken together, it appears that cognitive impairment is a major complication of CABG surgery, with impact in survivors well beyond the initial post-operative period.

### 4.1 Pre-operative cognitive impairment

The rate of pre-surgery impairment was heavily influenced by the method of classification utilized. When categorized by the 1SD method (where the pre-surgery cognition score of a patient is ≥1SD below study population/control patients/published norms) the presence of cognitive impairment was higher than when classified utilising a cut-off method (such as Mini-Mental State Examination (MMSE) ≤ 24) with rates of 32% and 14% respectively. This likely occurred as the 1SD method involves more comprehensive testing (neuropsychological test battery) allowing the assessment of subtle changes, compared to the commonly used screening tests administered in the cut-off method [16,[29], [30], [31]]. It is therefore likely that a third of patients present with cognitive impairment pre-operatively. This rate raises questions regarding patients' ability to appropriately provide informed consent, as cognitive impairment can decrease the ability to comprehend questions or instructions [32,33].

### 4.2 Delirium

The rate of delirium presence was heavily influenced by the method of diagnosis, where studies utilising a standardized diagnostic instrument (e.g. CAM/DRS) reported a clinically meaningful (confidence intervals did not overlap) higher pooled rate, compared to when no specific instrument was utilized (24% vs 11%). This highlights the importance of using a validated diagnostic instrument, such as the CAM, when screening for delirium presence so as not to overlook positive cases. Instruments such as the CAM–ICU, CAM and DRS can be conducted in <10-min, commonly taking around 5 min, deeming them feasible in a clinical setting. The development of delirium increases the length of hospital stay, risk of mortality within 6-months of surgery and is associated with long-term decreases in overall function, cognitive function and quality of life [[9], [10], [11], [12],^34^,^35^]. These outcomes not only impact patients themselves but also their families who both describe delirium as a horrendous, terrifying, shocking, daunting condition with long-term psychological consequences [36]. Therefore, screening for delirium in CABG patients post-operatively is integral as it may improve the patient's experience and possibly their long-term outcomes [37].

### 4.3 Post-operative cognitive impairment and decline

Cognitive impairment peaked at over 40% within the first four days following CABG, attenuated to 25% at around 1-year, and then rose again to around 40% from 1 to 5 years. In the long-term, >5-years following surgery, cognitive impairment was found in 16%, which is uncharacteristically low compared to other long term estimates and may be due to attrition and death of patients during follow-up. Combined, the two studies within the post 5-year analysis collected data on 285 of a possible 487 patients at follow-up. Of those patients whose data were not collected, 83 either died or were un-contactable and the remainder refused to participate [38,39]. In longitudinal studies, especially in older adults, returning participants have higher cognitive abilities compared to those who do not return [40]. Therefore, the sample utilized in these long-term follow-up analyses were most probably healthier (those still alive and healthy enough to participate) than those in the prior analyses (1–3 and 3–5 years), producing an underestimate of cognitive impairment.

Each time-point prevalence was greatly influenced by the method of classification used to define post-operative cognitive impairment (see Supplementary Table 3). Both the 1 SD and 20:20 methods resulted in higher prevalences of post-operative impairment across time-points compared to the RCI. An international consensus concerning the most appropriate and/or clinically relevant definition of how post-operative cognitive impairment should be classified, needs to be reached.

We suggest that future research investigating post-operative cognitive impairment and decline should utilize a RCI method for classification, with a neuropsychological battery of tests to improve the ability to track subtle change. The RCI method has demonstrated a good balance of both specificity and sensitivity [41,42], and attempts to eliminate practice effects of the conducted neuropsychology battery [[17], [18], [19], [20]]. This method takes into account individual change in comparison to the group, yet does not utilize arbitrary cut-offs like the 1 SD and 20:20 methods. Within this meta-analysis the RCI method revealed that cognitive impairment developed in over half (55%) of CABG patients acutely. This then decreased to 16% between 1 and 4-months, and increased back up to over a quarter (27%) between 1 and 3-years post-operatively. In the long-term, cognitive impairment was seen in just over a third of patients in >3 and 5-years post-CABG.

### 4.4 Dementia

Our findings indicate 7% of CABG patients develop dementia between 4.9 and 7.5 years post-surgery. This rate is similar to the global standard prevalence rate of approximately 4–9% in those ≥70 years old [43]. Yet, the data shows large heterogeneity driven by the extreme differences in reported incidence rates, ranging from 1.5 to 30.8%, between the three included studies. Studies reporting lower incidences of dementia, 1.5% [44] and 7% [45], had mean follow up times of <5 years post-surgery. The only study to follow-up at 7.5 years reported much higher rates, nearing 31% [38]. Additionally, the studies differed greatly in terms of diagnostic methods. Documented medical file codes (ICD-9) indicating diagnosed dementia were utilized by both studies reporting lower incidences [44,45]. The study reporting the highest incidence by Evered and colleagues [38], diagnosed dementia using a clinical dementia rating (conducted by an expert clinician) involving informant questionnaires, multiple neuropsychological tests including the MMSE, depression scores and also investigation into the patients instrumental activities of daily living. Therefore, perhaps more confidence should be placed in the study which used a more rigorous diagnostic method, and boasted the longest follow-up time, revealing 31% of CABG patients will develop dementia 7.5-years following surgery.

### 4.5 Strengths and limitations

This is the only comprehensive meta-analysis to investigate pooled estimates of cognitive impairment, including delirium and dementia, in CABG patients. The results of this meta-analysis have a pooled sample size of >90,000, which improves confidence and increases the generalizability of the results, but it is not without limitations.

This meta-analysis was limited to published studies that were written in English. Additionally, all analyses resulted in high heterogeneity demonstrating large variation across the literature. Research investigating cognitive outcomes following CABG has spanned across multiple decades, from the 1980s to the current 2010s. Additional analyses revealed that delirium estimates were greater in the 2000s and 2010s compared to past estimates, suggesting that knowledge surrounding delirium and screening for delirium has improved since the 1990s. Conversely, estimates of cognitive impairment in the 2010s were smaller compared to earlier decades, perhaps driven by improvements in surgical technology and techniques (see Supplementary Table 5 for decade analyses). This meta-analysis did not investigate the risk factors which contribute to the discussed post-operative outcomes. This was initially intended, however the very large number of studies identified in the search restricted this meta-analysis to only report on prevalence of cognitive outcomes.

## 5 Conclusions

A fifth of CABG patients have a cognitive impairment pre-surgery, which raises questions regarding their ability to provide informed consent. Post-surgery cognitive impairment increases to approximately 40% of patients acutely, decreases to approximately a fifth of patients up to 1-year post surgery and then increases to nearly 40% in the long-term (up to 5-years post-surgery). Additionally, we found that a quarter of CABG patients develop delirium post-operatively and that delirium presence is greatly under recognized when a diagnostic instrument is not utilized. Last, when a rigorous method is utilized for classifying dementia following CABG surgery it is reported that 31% of patients develop dementia.

It is critical to the future care of CABG patients that delirium is screened for in the post-operative setting, given one in four will likely experience delirium post-CABG. Correct diagnosis of delirium will improve the care of patients, in-turn perhaps decreasing the psychological demand that delirium can put on patients and their families. In regard to identifying cognitive impairment in a post-operative setting, the RCI method should be used in future research, as it displays the best specificity and sensitivity. Cognitive impairments are major complications of CABG surgery that cannot be overlooked. Adjustments are needed to clinical care to ensure that screening for impairments, including delirium, post-surgery are compulsory, so that patients are provided with the most appropriate care.

## Funding

DG is supported by Australian Government Research Training Program Scholarship. HADK is supported by a NHMRC-ARC Boosting Dementia Research Leadership Fellowship (GNT1135676). PJP is supported by a National Heart Foundation of Australia Future Leader Fellowship (FLF100412) and NHMRC-ARC Career Development Fellowship (GNT1161506). DD is supported by a Wellcome Trust Intermediate Clinical Fellowship (WT107467). AES is supported by an NHMRC-ARC Dementia Research Development Fellowship (GNT1097397). This project was supported by a National Heart Foundation of Australia Vanguard grant (101758 – VG 2017).

## Conflict of interest

The authors report no relationships that could be construed as a conflict of interest.
