# Supplementary material for: Cognitive outcomes following coronary artery bypass grafting: A systematic review and meta-analysis of 91,829 patients
Source: Int J Cardiol. 2019 Aug 15;289:43–9. doi: 10.1016/j.ijcard.2019.04.065 (PMC6548308; doi:10.1016/j.ijcard.2019.04.065)
Supplement: Supplementary material [file mmc1.docx]

Supplementary Figure 1

*Funnel plots of studies relative to outcome and time point.*

A

B

**Delirium**

**Pre-surgery**

**Immediate post-surgery (up to 4-days)**

**Post 5-days up to 1-month**

**Post 1-month to 4-months**

**Post 4-months to 6-months**

F

E

D

C

J

I

H

G

Note: Funnel plot could not be created for cognitive impairment time point analysis *post 5-years* due to there being an insufficient number of studies (i.e. N studies = 2)

**Post 6-months to 1-year**

**Post 1-year to 3-years**

**Post 3-years to 5-years**

**Dementia**

Supplementary Table 1

*Demographic Data, Statistical Analysis Inclusion and Quality Assessment Scores of Studies with Presence of Cognitive Impairment, Delirium and Dementia Before and After Coronary Artery Bypass Grafting*

|  |  |  |  |  |  |  | Cognitive Impairment Assessment Time Points | | | | | | | | |  |  |  |
| --- | --- | --- | --- | --- | --- | --- | --- | --- | --- | --- | --- | --- | --- | --- | --- | --- | --- | --- |
| Ref. No. | Lead Author, Year | Total No. of Patients | Total No. of Males | No. of pure CABG | Mean/Median Age | SD/Range of Age | T1 | T2 | T3 | T4 | T5 | T6 | T7 | T8 | T9 | Del | Dem | QA (/14) |
| [1] | Ahlgren, 2003 | 23 | 21 | 23 | 65.7 | 3.3 | - | - | - | x | - | - | - | - | - | - | - | 12 |
| [2] | Al Tmimi, 2016 | 92 | 78 | 92 | 67 | R: 46-86 | - | - | - | - | - | - | - | - | - | x | - | 13 |
| [3] | Alex, 2005 | 64 | 55 | 64 | 66.05 | 1.6 | - | - | - | x | - | - | - | - | - | - | - | 8 |
| [4] | Anastasiadis, 2011 | 60 | 56 | 60 | 65.29 | 8.23 | - | - | x | x | - | - | - | - | - | - | - | 11 |
| [5] | Andrew, 2001 | 59 | 48 | 59 | 65.1 | 10.9 | - | - | x | - | x | - | - | - | - | - | - | 10 |
| [6] | Arrowsmith, 1998 | 171 | 150 | 171 | 58.94 | 0.9 | - | - | - | x | - | - | - | - | - | - | - | 8 |
| [7] | Aykut, 2013 | 148 | 68 | 148 | 59.41 | 7.41 | x | - | - | x | - | - | - | - | - | - | - | 10 |
| [8] | Baba, 2007 | 218 | 152 | 218 | 71.25 | 5.5 | - | - | x | - | - | - | - | - | - | - | - | 11 |
| [9] | Bassano, 2016 | 366 | 307 | 366 | 67.28 | 9.43 | - | - | - | - | - | - | - | - | - | x | - | 7 |
| [10] | Bi, 2014 | 55 | 40 | 55 | 64.85 | 8.72 | - | - | x | - | - | - | - | - | - | - | - | 11 |
| [11] | Bily, 2015 | 500 | 337 | 216 | 65.05 | 10.4 | - | - | - | - | - | - | - | - | - | x | - | 12 |
| [12] | Bonacchi, 2006 | 42 | 29 | 42 | 63.59 | 6.78 | - | - | - | - | - | - | - | - | - | x | - | 7 |
| [13] | Boodhwani, 2006^††^ | 448 | 390 | 448 | 68.3 | 0.4 | - | - | x | - | - | - | - | - | - | - | - | 13 |
| [14] | Borger, 2001 | 83 | 67 | 83 | 60.28 | 9.36 | - | - | - | x | - | - | - | - | - | - | - | 9 |
| [15] | Borger, 2002 | 146 | N/A | 146 | N/A | N/A | - | - | x | - | - | - | - | - | - | - | - | 8 |
| [16] | Braekken, 1998 | 14 | 14 | 14 | N/A | N/A | - | - | - | x | - | - | - | - | - | - | - | 10 |
| [17] | Breu, 2015 | 467 | 350 | 326 | 66.85 | 10.33 | - | - | - | - | - | - | - | - | - | x | - | 12 |
| [18] | Breuer, 1983 | 421 | 361 | 421 | N/A | R: 46-85 | - | - | - | - | - | - | - | - | - | x | - | 12 |
| [19] | Brown, 2016 | 66 | 51 | 40 | 69.56 | 7.44 | - | - | - | - | - | - | - | - | - | x | - | 13 |
| [20] | Bruce, 2013 | 16 | 10 | 16 | 63.9 | 9 | - | - | x | x | - | - | - | - | - | - | - | 10 |
| [21] | Bucerius, 2004^*^ | 10759 | 8289 | 10759 | 65.01 | 9.44 | - | - | - | - | - | - | - | - | - | x | - | 7 |
| [22] | Bucerius, 2005^*^ | 9682 | 7500 | 9682 | N/A | N/A | - | - | - | - | - | - | - | - | - | x | - | 7 |
| [23] | Butterworth, 1999 | 67 | 45 | 67 | 66 | 6.69 | - | - | x | x | - | - | - | - | - | - | - | 7 |
| [24] | Butterworth, 2005 | 381 | 308 | 381 | N/A | N/A | - | - | x | x | x | - | - | - | - | - | - | 6 |
| [25] | Buziashvili, 2005 | 40 | 40 | 40 | 52.6 | 6.4 | - | - | x | - | - | - | - | - | - | - | - | 9 |
| [26] | Chakravarthy, 2008 | 114 | N/A | 114 | N/A | N/A | x | - | x | x | x | - | - | - | - | - | - | 7 |
| [27] | Chernov, 2006 | 65 | 62 | 65 | 54.34 | 4.68 | - | - | x | - | x | - | - | - | - | - | - | 7 |
| [28] | Christiansen, 2016 | 8 | 7 | 8 | 63.4 | 10.7 | - | x | - | - | - | - | - | - | - | - | - | 4 |
| [29] | Colak, 2015 | 190 | 148 | 190 | 62.66 | 7.96 | - | - | x | - | - | - | - | - | - | x | - | 7 |
| [30] | Cook, 2007 | 27 | N/A | 27 | 72 | N/A | - | - | x | x | - | - | - | - | - | - | - | 7 |
| [31] | Cumurcu, 2008 | 50 | 37 | 50 | 59.62 | 10.66 | - | - | - | - | - | - | - | - | - | x | - | 9 |
| [32] | Dabrowski, 2010 | 92 | 73 | 92 | 66 | 6 | - | x | - | - | - | - | - | - | - | - | - | 11 |
| [33] | deTournay-Jette, 2011 | 61 | 51 | 61 | 70.39 | 4.69 | - | - | x | x | - | - | - | - | - | - | - | 12 |
| [34] | deBaar, 2003 | 60 | 46 | 60 | 66.48 | 8.52 | - | - | x | - | - | - | - | - | - | - | - | 11 |
| [35] | deTournay-Jette, 2012 | 51 | 41 | 51 | 70.65 | 4.42 | - | - | x | x | - | - | - | - | - | - | - | 11 |
| [36] | Dieleman, 2009 | 281 | 192 | 281 | 61.3 | 9 | - | - | - | - | - | x | - | - | - | - | - | 12 |
| [37] | Djaiani, 2007 | 226 | 203 | 226 | 67.25 | 6.05 | - | - | - | x | - | - | - | - | - | - | - | 9 |
| [38] | Djaiani, 2008 | 113 | 83 | 113 | 73.95 | 3.45 | - | - | - | - | - | - | - | - | - | x | - | 7 |
| [39] | Djaiani, 2012 | 170 | 153 | 170 | 66.95 | 5.95 | - | - | - | - | - | x | - | - | - | - | - | 9 |
| [40] | Dong, 2014 | 108 | 83 | 108 | 63 | 7.9 | - | - | x | - | - | - | - | - | - | x | - | 9 |
| [41] | Dowd, 2001 | 78 | 56 | 78 | 70.8 | 3 | - | x | - | - | - | - | - | - | - | - | - | 11 |
| [42] | Dumas, 1999 | 47 | 36 | 47 | 59.75 | 8.69 | - | - | x | x | - | - | - | - | - | - | - | 9 |
| [43] | Ebert, 2001 | 42 | 25 | 42 | 63.1 | R: 40-80 | - | x | x | - | - | - | - | - | - | x | - | 11 |
| [44] | Eriksson, 2002 | 52 | 40 | 52 | 70.27 | 5.53 | - | - | - | - | - | - | - | - | - | x | - | 13 |
| [45] | Eryomina, 2015 | 74 | N/A | 74 | 60.25 | 6.42 | x | - | x | - | - | - | - | - | - | - | - | 8 |
| [46] | Evered, 2009 | 332 | 255 | 332 | 68 | 7.6 | x | - | - | x | - | x | - | - | - | - | - | 9 |
| [47] | Evered, 2010 | 296 | 225 | 296 | 68.2 | 7.6 | - | - | x | - | - | - | - | - | - | - | - | 10 |
| [48] | Evered, 2011 | 312 | 242 | 312 | 67.78 | 7.51 | - | - | x | x | - | - | - | - | - | - | - | 11 |
| [49] | Evered, 2016 | 326 | 252 | 326 | 67.96 | 7.62 | x | - | - | - | - | - | - | - | x | - | x | 11 |
| [50] | Farhoudi, 2010 | 154 | 123 | 154 | 57.17 | 9.75 | - | - | - | x | - | - | - | - | - | - | - | 8 |
| [51] | Forrest, 2011 | 28 | 25 | 28 | 60.2 | 1.7 | - | - | x | x | - | - | - | - | - | - | - | 7 |
| [52] | Gasparovic, 2013 | 59 | 49 | 59 | 65.37 | 8.63 | - | - | - | - | - | - | - | - | - | x | - | 8 |
| [53] | Ghafari, 2012 | 106 | 66 | 106 | 58.5 | N/A | - | - | x | x | - | - | - | - | - | - | - | 11 |
| [54] | Goto, 2000^†^ | 177 | 117 | 177 | 70.26 | 4.99 | - | x | x | - | - | - | - | - | - | - | - | 13 |
| [55] | Goto, 2001^†^ | 421 | 289 | 421 | 70 | 5.4 | - | - | x | - | - | - | - | - | - | - | - | 13 |
| [56] | Goto, 2003^†^ | 463 | 318 | 463 | 70 | 5.48 | - | - | x | - | - | - | - | - | - | - | - | 11 |
| [57] | Grigore, 2002 | 165 | 117 | 165 | 61.35 | 10.56 | - | - | - | x | - | - | - | - | - | - | - | 9 |
| [58] | Grocott, 2002 | 300 | 252 | 300 | 61 | 10 | - | - | - | x | - | - | - | - | - | - | - | 10 |
| [59] | Gunaydin, 1998 | 50 | 34 | 50 | 49.2 | 7.7 | - | - | - | - | - | - | - | - | - | x | - | 11 |
| [60] | Habib, 2014 | 134 | 113 | 134 | 53.7 | 8.36 | x | - | x | - | x | - | - | - | - | - | - | 11 |
| [61] | Haljan,2009 | 32 | 32 | 32 | 60 | 7.2 | - | - | x | x | - | - | - | - | - | - | - | 12 |
| [62] | Hall, 1999 | 35 | 27 | 35 | 65.9 | 9.1 | - | - | x | - | - | - | - | - | - | - | - | 13 |
| [63] | Hall, 2005 | 45 | 36 | 45 | 63.9 | 9.3 | - | - | x | - | - | - | - | - | - | - | - | 11 |
| [64] | Hammon, 2006 | 237 | 178 | 237 | 63.61 | 8.87 | - | x | - | x | x | - | - | - | - | - | - | 8 |
| [65] | Harmon, 2004 | 35 | 28 | 35 | 61.7 | 7.51 | - | x | - | x | - | - | - | - | - | x | - | 11 |
| [66] | Harmon, 2005 | 36 | 30 | 36 | 64.07 | N/A | - | x | - | x | - | - | - | - | - | x | - | 11 |
| [67] | Harrison, 1989^\|\|\|\|^ | 47 | N/A | 47 | 55.14 | 7.95 | - | - | x | x | - | - | - | - | - | - | - | 7 |
| [68] | Hernandez, 2007 | 201 | 161 | 201 | N/A | N/A | - | x | - | - | x | - | - | - | - | - | - | 10 |
| [69] | Herrmann, 2000 | 55 | N/A | 55 | 59.6 | 9.23 | - | - | - | - | - | - | - | - | - | x | - | 8 |
| [70] | Heyer, 1997 | 99 | 81 | 99 | 63.92 | 11.1 | - | - | x | x | - | - | - | - | - | - | - | 8 |
| [71] | Ho, 2004 | 1677 | N/A | 1677 | N/A | N/A | - | - | - | - | x | - | - | - | - | - | - | 8 |
| [72] | Hudetz, 2011 | 22 | 22 | 22 | 70 | 8 | - | - | x | - | - | - | - | - | - | x | - | 10 |
| [73] | Humphreys, 2016 | 173 | 148 | 173 | 63.47 | 10.1 | - | - | - | - | - | - | - | - | - | x | - | 8 |
| [74] | Ito, 2012 | 449 | 308 | 449 | 70 | 5.42 | x | - | - | - | - | - | - | - | - | - | - | 8 |
| [75] | Jensen, 2006 | 120 | 72 | 120 | 75.51 | 4.51 | - | - | - | x | - | - | - | - | - | - | - | 11 |
| [76] | Jensen, 2008 | 90 | 55 | 90 | 75.26 | 4.67 | - | - | - | - | - | x | - | - | - | - | - | 12 |
| [77] | Jonsson, 2004 | 56 | 47 | 56 | 60.4 | 9 | - | - | x | - | - | - | - | - | - | - | - | 12 |
| [78] | Joung, 2013 | 70 | 57 | 70 | 60.05 | 7.8 | - | - | x | - | - | - | - | - | - | - | - | 11 |
| [79] | Kadoi, 2001 | 185 | 138 | 185 | N/A | N/A | - | - | x | - | x | - | - | - | - | - | - | 9 |
| [80] | Kadoi, 2002 | 60 | 53 | 60 | 62.75 | 8.5 | - | - | - | x | - | - | - | - | - | - | - | 11 |
| [81] | Kadoi, 2003 | 180 | 136 | 180 | 65 | 9 | - | - | - | - | x | - | - | - | - | - | - | 11 |
| [82] | Kadoi, 2005^‡‡^ | 280 | 210 | 280 | 65.07 | 9.93 | - | - | x | - | x | - | - | - | - | - | - | 11 |
| [83] | Kadoi, 2007 | 106 | 53 | 106 | 62.55 | 10.45 | - | - | - | - | x | - | - | - | - | - | - | 11 |
| [84] | Kadoi, 2011 (a)^‡^ | 90 | 68 | 90 | 65 | 9 | - | - | x | - | x | - | - | - | - | - | - | 11 |
| [85] | Kadoi, 2011 (b)^‡^ | 124 | 89 | 124 | 61.29 | 5.39 | - | - | x | - | x | - | - | - | - | - | - | 11 |
| [86] | Kadoi, 2013^‡‡^ | 97 | 75 | 97 | 61.01 | 6 | - | - | x | - | x | - | - | - | - | - | - | 11 |
| [87] | Kara, 2015 | 79 | 62 | 79 | 60.06 | 9.81 | x | - | x | - | - | - | - | - | - | - | - | 13 |
| [88] | Kaukinen, 2000 | 36 | 31 | 36 | 58.1 | 7.45 | - | - | x | - | - | - | x | - | - | - | - | 10 |
| [89] | Kazmierski, 2014 (a)^§^ | 113 | 90 | 113 | 64 | R: 59-71 | x | - | - | - | - | - | - | - | - | x | - | 13 |
| [90] | Kazmierski, 2014 (b)^§^ | 102 | N/A | 102 | N/A | N/A | - | - | - | - | - | - | - | - | - | x | - | 13 |
| [91] | Keizer, 2005 | 281 | 191 | 281 | 61.3 | 9 | - | - | - | x | - | - | - | - | - | - | - | 7 |
| [92] | Kempfert, 2008 | 99 | 78 | 99 | 74.99 | 0.55 | - | - | - | - | - | - | - | - | - | x | - | 10 |
| [93] | Khan, 2014 | 735 | 577 | 735 | 55.64 | 9.65 | - | - | - | - | - | - | - | - | - | x | - | 11 |
| [94] | Khatri, 1999 | 170 | 127 | 170 | 61 | 10 | - | - | - | x | - | - | - | - | - | - | - | 9 |
| [95] | Knipp, 2008 | 39 | 31 | 39 | 67 | 9.8 | - | - | x | x | - | - | x | - | - | - | - | 12 |
| [96] | Kok, 2014 | 59 | 53 | 59 | 62.8 | 9.4 | - | x | - | x | - | - | - | - | - | - | - | 12 |
| [97] | Kok, 2017 | 57 | N/A | 57 | N/A | N/A | - | - | - | - | - | - | x | - | - | - | - | 8 |
| [98] | Kozora, 2010 | 1156 | 1149 | 1156 | 61.95 | 8.15 | - | - | - | - | - | x | - | - | - | - | - | 11 |
| [99] | Krzych, 2014 | 5781 | 4031 | 4076 | N/A | N/A | - | - | - | - | - | - | - | - | - | x | - | 9 |
| [100] | Lahariya, 2014 | 309 | N/A | 15 | 58.24 | 12.9 | - | - | - | - | - | - | - | - | - | x | - | 11 |
| [101] | Leacche, 2003 | 640 | 467 | 640 | 63.74 | 10.39 | - | - | - | - | - | - | - | - | - | x | - | 7 |
| [102] | Lee, 2003 | 60 | 46 | 60 | 65.75 | 10.4 | - | - | x | - | - | x | - | - | - | - | - | 9 |
| [103] | Lee, 2005 | 5216 | 5124 | 5216 | 67.2 | 6.7 | - | - | - | - | - | - | - | - | - | - | x | 10 |
| [104] | Lelis, 2006 | 87 | 68 | 87 | 64.8 | 9.46 | - | x | x | - | - | - | - | - | - | - | - | 13 |
| [105] | Lewis, 2006 (a)^§§^ | 204 | 152 | 204 | 67.8 | 7.9 | - | - | x | - | - | - | - | - | - | - | - | 9 |
| [106] | Lewis, 2006 (b)^§§^ | 204 | 152 | 204 | 67.8 | 7.9 | - | - | x | x | - | - | - | - | - | - | - | 10 |
| [107] | Lewis, 2007^§§^ | 204 | 152 | 204 | 68.8 | 7 | - | - | x | - | - | - | - | - | - | - | - | 11 |
| [108] | Li, 2015 | 38 | 34 | 38 | 62.4 | 11.8 | - | - | - | - | - | - | - | - | - | x | - | 10 |
| [109] | Liu, 2009 | 227 | 209 | 227 | 60 | 8 | - | - | x | x | - | - | - | - | - | - | - | 11 |
| [110] | Loponen, 2008 | 300 | 237 | 300 | 66.17 | 8.89 | - | - | - | - | - | - | - | - | - | x | - | 9 |
| [111] | Lund, 2003 | 52 | 43 | 52 | 62.88 | N/A | - | - | - | x | - | - | - | - | - | - | - | 8 |
| [112] | Lund, 2005 | 120 | 94 | 120 | 65 | 8.1 | - | - | - | x | - | x | - | - | - | - | - | 11 |
| [113] | Maekawa, 2011 | 362 | 221 | 156 | 69.5 | 9.7 | x | - | x | - | - | - | - | - | - | - | - | 12 |
| [114] | Mahanna, 1996 | 232 | 186 | 232 | 61 | 10 | - | - | x | x | x | - | - | - | - | - | - | 8 |
| [115] | Mardani, 2012 | 196 | 183 | 174 | 61.84 | 11.83 | - | - | - | - | - | - | - | - |  | x | - | 10 |
| [116] | Mariscalco, 2012 | 4079 | 3220 | 2472 | 67.8 | 9.2 | - | - | - | - | - | - | - | - | - | x | - | 13 |
| [117] | Martens, 2004 | 77 | 55 | 77 | 66.95 | 7.8 | - | - | x | - | - | - | - | - | - | - | - | 12 |
| [118] | Martin, 2010^\|\|^ | 14262 | 10912 | 14262 | N/A | N/A | - | - | - | - | - | - | - | - | - | x | - | 6 |
| [119] | Martin, 2012^\|\|^ | 8474 | 6391 | 8474 | N/A | N/A | - | - | - | - | - | - | - | - | - | x | - | 9 |
| [120] | Mathew, 2003 | 460 | 292 | 460 | 62.69 | 11.4 | - | - | - | x | - | - | - | - | - | - | - | 11 |
| [121] | Mathew, 2005 | 418 | 285 | 418 | 61.25 | 10.38 | - | - | - | x | - | - | - | - | - | - | - | 10 |
| [122] | Mathew, 2006 | 121 | N/A | 121 | N/A | N/A | - | - | - | x | - | - | - | - | - | - | - | 10 |
| [123] | Mathew, 2007 | 677 | 471 | 677 | 61.7 | 10.5 | - | - | - | x | - | - | - | - | - | - | - | 11 |
| [124] | Millar, 2001 | 120 | 92 | 120 | N/A | N/A | x | - | x | - | x | - | - | - | - | - | - | 7 |
| [125] | Miyazaki, 2011 | 768 | N/A | 768 | N/A | N/A | - | - | - | - | - | - | - | - | - | x | - | 5 |
| [126] | Molstrom, 2016 | 10 | 9 | 10 | N/A | N/A | - | x | - | - | - | - | - | - | - | - | - | 10 |
| [127] | Mongero, 2001 | 39 | N/A | 39 | N/A | N/A | - | - | x | x | - | - | - | - | - | - | - | 4 |
| [128] | Mu, 2010 | 243 | 200 | 243 | 61 | 8.3 | - | - | - | - | - | - | - | - | - | x | - | 13 |
| [129] | Mu, 2013 | 166 | 141 | 166 | 60 | 8.9 | x | - | x | - | - | - | - | - | - | - | - | 13 |
| [130] | Mullges, 2002 | 91 | 80 | 91 | 60.86 | 8.28 | - | - | x | - | - | - | - | x | - | - | - | 10 |
| [131] | Murkin, 1995 | 316 | 264 | 316 | 60.9 | 8.3 | - | - | x | x | - | - | - | - | - | - | - | 10 |
| [132] | Mutch, 2011 | 1124 | 880 | 1124 | 66.9 | N/A | - | - | - | - | - | - | - | - | - | - | x | 10 |
| [133] | Nathan, 2007 | 223 | 189 | 223 | 67.91 | 5.41 | - | - | - | - | - | - | - | x | - | - | - | 11 |
| [134] | Newman, 2001 | 261 | 195 | 261 | 60.9 | 10.6 | - | - | x | x | x | - | - | x | - | - | - | 10 |
| [135] | Nikolic, 2012 | 370 | 271 | 370 | N/A | N/A | - | - | - | - | - | - | - | - | - | x | - | 8 |
| [136] | Norkiene, 2007 | 1367 | 1035 | 1367 | 64.98 | 9.14 | - | - | - | - | - | - | - | - | - | x | - | 11 |
| [137] | Norkiene, 2011 | 127 | 103 | 127 | 60.91 | 7.24 | - | - | x | - | - | - | - | - | - | x | - | 10 |
| [138] | Oh, 2008 | 46 | 36 | 46 | 63 | 5.5 | - | x | x | - | - | - | - | - | - | - | - | 11 |
| [139] | Oldham, 2015 | 102 | 76 | 102 | 65.1 | 9 | x | - | - | - | - | - | - | - | - | x | - | 11 |
| [140] | Omiya, 2015 | 88 | N/A | 88 | 69 | 7 | - | - | - | - | - | - | - | - | - | x | - | 11 |
| [141] | Osse, 2012 | 125 | 82 | 55 | 75.84 | 3.47 | - | - | - | - | - | - | - | - | - | x | - | 13 |
| [142] | Otomo, 2013 | 153 | 109 | 153 | 72 | 7 | - | - | - | - | - | - | - | - | - | x | - | 13 |
| [143] | Ozturk, 2016 | 40 | 31 | 40 | 56.13 | 5.59 | - | x | - | - | - | - | - | - | - | - | - | 13 |
| [144] | Palmbergen, 2012 | 642 | 452 | 497 | 68.5 | 9.79 | - | - | - | - | - | - | - | - | - | x | - | 12 |
| [145] | Patel, 1996 | 70 | 66 | 70 | 57.25 | N/A | - | - | - | x | - | - | - | - | - | - | - | 11 |
| [146] | Peterson, 2012 | 248 | 198 | 248 | 65.81 | 9.35 | - | - | - | - | x | - | - | - | - | - | - | 10 |
| [147] | Phillips-Bute, 2006 | 732 | 462 | 732 | 61.93 | 11.17 | - | - | - | x | - | x | - | - | - | - | - | 7 |
| [148] | Prakanrattana, 2007 | 126 | 74 | 90 | 61.06 | 9.52 | - | - | - | - | - | - | - | - | - | x | - | 13 |
| [149] | Ramlawi, 2006 | 40 | 35 | 30 | 67.3 | 10.5 | - | x | - | - | - | - | - | - | - | - | - | 10 |
| [150] | Rasmussen, 1999 | 35 | 26 | 35 | Med: 70 | R: 61-80 | - | - | x | x | - | - | - | - | - | - | - | 11 |
| [151] | Rasmussen, 2002 | 15 | 15 | 15 | Med: 65 | R: 51-77 | - | - | x | x | - | - | - | - | - | - | - | 10 |
| [152] | Raymond, 2006 | 55 | 48 | 55 | 64.4 | 9 | - | - | x | - | - | - | - | - | - | - | - | 11 |
| [153] | Reents, 2002 | 47 | 41 | 47 | 60.4 | 8 | - | - | x | - | - | - | - | - | - | x | - | 9 |
| [154] | Restrepo, 2002 | 13 | 10 | 13 | 65 | 9 | - | - | x | - | - | - | - | - | - | - | - | 10 |
| [155] | Ringaitiene, 2015 | 99 | 70 | 99 | 67.6 | 7.78 | - | - | - | - | - | - | - | - | - | x | - | 11 |
| [156] | Robson, 2000 | 124 | N/A | 124 | 59.44 | 9.25 | - | - | - | x | - | - | - | - | - | - | - | 8 |
| [157] | Rolfson, 1999 | 71 | 57 | 71 | 71 | N/A | - | - | - | - | - | - | - | - | - | x | - | 12 |
| [158] | Royse, 2000 | 47 | 37 | 47 | 64.22 | 1.78 | - | - | x | x | - | - | - | - | - | - | - | 9 |
| [159] | Royse, 2011 | 180 | 153 | 180 | 62.79 | 10.5 | - | - | x | x | - | - | - | - | - | x | - | 9 |
| [160] | Rudolph, 2005 | 36 | 36 | 36 | 68.8 | 9.2 | - | - | - | - | - | - | - | - | - | x | - | 13 |
| [161] | Rudolph, 2006 | 80 | 62 | 69 | 74.9 | 6.2 | - | - | - | - | - | - | - | - | - | x | - | 10 |
| [162] | Rudolph, 2009 | 68 | 67 | 68 | 70.7 | 8.2 | - | - | - | - | - | - | - | - | - | x | - | 13 |
| [163] | Saczynski, 2012 | 225 | 171 | 176 | 73 | 6.7 | - | - | - | - | - | - | - | - | - | x | - | 10 |
| [164] | Santos, 2005 | 220 | 142 | 220 | 71.2 | 5.8 | - | - | - | - | - | - | - | - | - | x | - | 10 |
| [165] | Scott, 2002 | 103 | 84 | 103 | 64.77 | 1.3 | - | - | x | - | - | - | - | - | - | - | - | 12 |
| [166] | Sellman, 1993 | 54 | 54 | 54 | 59 | R: 44-69 | - | - | - | x | x | - | - | - | - | - | - | 11 |
| [167] | Selnes, 2008 | 152 | 115 | 152 | 63.6 | 9.4 | - | - | - | - | - | - | - | - | x | - | - | 10 |
| [168] | Sevuk, 2015 | 200 | 128 | 200 | 70.65 | 3.95 | - | - | - | - | - | - | - | - | - | x | - | 11 |
| [169] | Shaw, 1986 | 312 | 276 | 312 | 53.4 | 7.4 | - | - | x | - | - | - | - | - | - | - | - | 11 |
| [170] | Shaw, 1987 | 259 | 234 | 259 | 53.5 | R: 33-70 | - | - | - | - | x | - | - | - | - | - | - | 10 |
| [171] | Shioiri, 2016 | 84 | 52 | 38 | 70.24 | 6.48 | - | - | - | - | - | - | - | - | - | x | - | 11 |
| [172] | Siepe, 2011 | 92 | 74 | 92 | 66.87 | 8.98 | - | - | - | - | - | - | - | - | - | x | - | 13 |
| [173] | Silbert, 2004 | 50 | 41 | 50 | 66.3 | R: 55-82 | - | x | - | - | - | - | - | - | - | - | - | 12 |
| [174] | Silva, 2016 | 88 | 68 | 88 | 61.8 | 9.2 | - | - | x | - | - | x | - | - | - | x | - | 10 |
| [175] | Sirvinskas, 2014 | 50 | 37 | 50 | 62.6 | 5.1 | - | - | x | - | - | - | - | - | - | - | - | 11 |
| [176] | Slater, 2009 | 240 | 201 | 240 | 64.74 | 9.96 | - | - | x | x | - | - | - | - | - | x | - | 6 |
| [177] | Smith, 1986^\|\|\|\|^ | 55 | 51 | 55 | 54.7 | R: 37-74 | - | - | x | x | - | - | - | - | - | - | - | 7 |
| [178] | Smith, 1988^\|\|\|\|^ | 76 | 70 | 76 | 55.1 | R: 37-71 | - | - | x | x | - | x | - | - | - | - | - | 7 |
| [179] | Soehle, 2015 | 81 | 57 | 44 | 72.9 | 6.2 | - | - | - | - | - | - | - | - | - | x | - | 11 |
| [180] | Song, 2016 | 813 | 639 | 813 | 62.18 | 8.14 | - | - | x | - | - | - | - | - | - | - | - | 11 |
| [181] | Stanley, 2002 | 308 | 212 | 308 | 61.34 | 10.78 | - | - | - | x | - | - | - | - | - | - | - | 9 |
| [182] | Stroobant, 2002^#^ | 49 | 44 | 49 | 58.78 | 7.62 | - | - | x | - | x | - | - | - | - | - | - | 8 |
| [183] | Stroobant, 2005^#^ | 50 | 46 | 50 | 59.89 | 7.49 | - | - | x | - | x | - | - | - | - | - | - | 8 |
| [184] | Stroobant, 2008 | 54 | 48 | 54 | 59 | 7.5 | - | - | - | - | - | - | - | x | - | - | - | 10 |
| [185] | Suksompong, 2002 | 110 | 110 | 110 | 61.95 | 7.58 | - | x | - | - | - | - | - | - | - | - | - | 11 |
| [186] | Svenmarker, 2004 | 60 | 60 | 60 | 59.5 | 1.1 | - | x | - | - | - | - | - | - | - | - | - | 10 |
| [187] | Swaminathan, 2002 | 282 | 201 | 282 | 61 | 10.44 | - | - | - | x | - | - | - | - | - | - | - | 10 |
| [188] | Sylivris, 1998 | 41 | 31 | 41 | 69.8 | 6.9 | - | - | x | - | - | - | - | - | - | - | - | 10 |
| [189] | Szwed, 2014 | 74 | 51 | 74 | 64.03 | 3.28 | - | - | x | - | - | - | - | - | - | - | - | 12 |
| [190] | Tabatabaie, 2015 | 60 | 60 | 60 | 65 | 5.3 | - | - | - | - | - | - | - | - | - | x | - | 13 |
| [191] | Tagarakis, 2007 | 137 | 99 | 137 | 69.55 | 7.63 | - | - | - | - | - | - | - | - | - | x | - | 8 |
| [192] | Taggart, 2003 | 150 | 131 | 150 | 62.3 | 8.7 | - | - | x | x | - | - | - | - | - | - | - | 8 |
| [193] | Tan, 2008 | 53 | N/A | 17 | 62.7 | 8.52 | - | - | - | - | - | - | - | - | - | x | - | 12 |
| [194] | Thornton, 2005 | 71 | 71 | 71 | 61 | 8.5 | - | - | - | x | x | - | - | - | - | - | - | 6 |
| [195] | Toeg, 2013^††^ | 652 | 576 | 652 | 64.37 | 9 | - | - | x | x | - | - | - | - | - | - | - | 9 |
| [196] | Toner, 1994 | 15 | 12 | 15 | 59 | R: 43-69 | - | - | - | x | - | - | - | - | - | - | - | 10 |
| [197] | Toner, 1997^**^ | 61 | 57 | 61 | 59 | R: 43-73 | - | - | - | x | - | - | - | - | - | - | - | 7 |
| [198] | Toner, 1998^**^ | 62 | 55 | 62 | 60.3 | 8.7 | - | - | x | x | - | - | - | - | - | - | - | 9 |
| [199] | Trubnikova, 2014 | 101 | 101 | 101 | 56.6 | 5.85 | - | - | x | - | - | x | - | - | - | - | - | 7 |
| [200] | Tse, 2015 | 679 | 500 | 396 | 66.96 | 11.72 | - | - | - | - | - | - | - | - | - | x | - | 10 |
| [201] | Tully, 2010 | 158 | 125 | 140 | 64.68 | 10.56 | - | - | - | - | - | - | - | - | - | x | - | 10 |
| [202] | Vanninen, 1998 | 38 | 32 | 38 | 64 | R: 55-73 | - | - | - | x | - | - | - | - | - | - | - | 9 |
| [203] | van Dijk, 2002 | 281 | 193 | 281 | 61.25 | 9 | - | - | - | - | - | x | - | - | - | - | - | 11 |
| [204] | van Dijk, 2004 | 281 | 191 | 281 | 61.2 | 9 | - | x | - | - | - | - | - | - | - | - | - | 13 |
| [205] | van Dijk, 2007 | 281 | 192 | 281 | 61.2 | 9 | - | - | - | - | - | - | - | x | - | - | - | 14 |
| [206] | Vedin, 2006 | 70 | 56 | 70 | 65 | N/A | - | - | x | x | x | - | - | - | - | - | - | 10 |
| [207] | Walzer, 1997 | 70 | 59 | 70 | 60.7 | R: 45-78 | - | - | - | - | - | - | - | - | - | x | - | 9 |
| [208] | Wang, 2002 | 88 | 86 | 88 | 58.57 | 9.55 | - | - | x | - | - | - | - | - | - | - | - | 13 |
| [209] | Whitaker, 2004 | 198 | 170 | 198 | 64.1 | 8.7 | - | - | - | x | - | - | - | - | - | - | - | 11 |
| [210] | Yilmaz, 2016 | 137 | 105 | 137 | 61.02 | 7.83 | - | - | - | - | - | - | - | - | - | x | - | 10 |
| [211] | Yoda, 2004 | 10 | 4 | 10 | 63.4 | 5.5 | - | - | x | - | - | - | - | - | - | - | - | 10 |
| [212] | Yoon, 2001 | 201 | 154 | 201 | 50.5 | 8 | - | - | - | - | - | - | - | - | - | x | - | 8 |
| [213] | Zamvar, 2002 | 60 | 52 | 60 | 62.55 | 9.55 | - | - | x | x | - | - | - | - | - | - | - | 11 |
| [214] | Zhang, 2015 | 249 | 197 | 201 | 62.9 | 9.34 | - | - | - | - | - | - | - | - | - | x | - | 13 |
| [215] | Zimpfer, 2004 | 104 | 93 | 104 | 64.1 | 9.8 | - | - | x | x | - | - | x | - | - | - | - | 8 |
| ^*, †, ‡, §, \|\|, #, **^ Suspected overlap of samples; ^††, ‡‡, §§, \|\|\|\|^ Known overlap of samples | | | | | | | | | | | | | | | | | | |
| Ref No. = supplementary reference list number; Del = delirium; Dem = dementia; Med = median; QA = quality assessment; R = range; SD = standard deviation; T = time; T1 = baseline (pre-surgery); T2 = Immediate post-surgery (immediate to 4 days); T3 = 5 days to 1 month (including discharge and non-inclusive of 1 month); T4 = 1 month to 4 months (inclusive); T5 = post 4 months to 6 months (inclusive); T6 = post 6 months to 1 year (inclusive); T7 = post 1 year to 3 years (inclusive); T8 = post 3 years to 5 years (inclusive); T9 = post 5 years | | | | | | | | | | | | | | | | | | |

Supplementary Table 2: Pooled estimates relative to outcome (dementia, delirium and cognitive impairment), classification method and time point.

|  |  | Pooled-effect (prevalence) | | | | | | Heterogeneity | | | |
| --- | --- | --- | --- | --- | --- | --- | --- | --- | --- | --- | --- |
|  |  | N studies | N participants | Point-estimate | 95%CI | Z-value | p | Q-value | df(Q) | p | I^2^ |
| Dementia | Averaged over all dementia classifications | 3 | 6457 | 0.07 | 0.01-0.30 | -2.93 | .003 | 240.06 | 2 | <.001 | 99.17 |
| Delirium | Averaged over all delirium classifications | 70 | 61116 | 0.18 | 0.15-0.21 | -14.46 | <.001 | 2876.12 | 69 | <.001 | 97.60 |
|  | Plus standardised measurement tool | 39 | 7660 | 0.24 | 0.19-0.31 | -7.08 | <.001 | 811.18 | 38 | <.001 | 95.32 |
|  | No tool used | 31 | 53456 | 0.11 | 0.09-0.13 | -19.81 | <.001 | 793.63 | 30 | <.001 | 96.22 |
| Pre-surgery | Averaged over all CI classifications | 13 | 2274 | 0.19 | 0.13-0.26 | -6.71 | <.001 | 152.16 | 12 | <.001 | 92.03 |
|  | 1 SD method^†^ | 4 | 885 | 0.32 | 0.19-0.48 | -2.14 | .032 | 53.29 | 3 | <.001 | 94.37 |
|  | 20% method | - | - | - | - | - | - | - | - | - | - |
|  | RCI method | - | - | - | - | - | - | - | - | - | - |
|  | Cutoff method^††^ | 9 | 1376 | 0.14 | 0.09-0.21 | -7.27 | <.001 | 57.25 | 8 | <.001 | 86.03 |
| Immediate post-surgery (up to 4-days) | Averaged over all CI classifications | 19 | 1542 | 0.43 | 0.35-0.52 | -1.47 | .143 | 175.05 | 18 | <.001 | 89.72 |
|  | 1 SD method | 3 | 132 | 0.60 | 0.40-0.77 | 0.99 | .320 | 9.30 | 2 | .010 | 78.50 |
|  | 20% method | 5 | 551 | 0.48 | 0.37-0.59 | -0.40 | .690 | 18.18 | 4 | <.001 | 78.00 |
|  | RCI method | 4 | 180 | 0.55 | 0.42-0.68 | 0.74 | .457 | 8.69 | 3 | .034 | 65.49 |
|  | Cutoff method | 4 | 366 | 0.27 | 0.18-0.37 | -4.17 | <.001 | 9.21 | 3 | .027 | 67.42 |
| Post 5-days up to 1-month | Averaged over all CI classifications | 88 | 11065 | 0.39 | 0.35-0.44 | -4.28 | <.001 | 1618.48 | 87 | <.001 | 94.62 |
|  | 1 SD method | 30 | 3865 | 0.41 | 0.35-0.48 | -2.59 | .097 | 356.00 | 29 | <.001 | 91.85 |
|  | 20% method | 30 | 2727 | 0.44 | 0.36-0.53 | -1.21 | 0.228 | 455.50 | 29 | <.001 | 93.63 |
|  | RCI method | 12 | 1180 | 0.38 | 0.33-0.44 | -4.19 | <.001 | 30.06 | 11 | .002 | 63.40 |
|  | Cutoff method | 11 | 2485 | 0.15 | 0.11-0.20 | -9.63 | <.001 | 69.24 | 10 | <.001 | 85.56 |
| Post 1-month to 4-months | Averaged over all CI classifications | 71 | 9658 | 0.25 | 0.22- 0.28 | -13.22 | <.001 | 650.36 | 70 | <.001 | 89.24 |
|  | 1 SD method | 33 | 5832 | 0.28 | 0.24- 0.33 | -8.15 | <.001 | 400.14 | 32 | <.001 | 92.00 |
|  | 20% method | 18 | 1885 | 0.26 | 0.21-0.31 | -7.79 | <.001 | 82.29 | 17 | <.001 | 79.34 |
|  | RCI method | 12 | 1231 | 0.16 | 0.10-0.24 | -6.42 | <.001 | 79.81 | 11 | <.001 | 86.22 |
|  | Cutoff method | 4 | 522 | 0.11 | 0.04-0.25 | -4.07 | <.001 | 32.78 | 3 | <.001 | 90.85 |
| Post 4-months to 6-months | Averaged over all CI classifications | 25 | 3967 | 0.19 | 0.15-0.24 | -9.93 | <.001 | 223.56 | 24 | <.001 | 89.26 |
|  | 1 SD method | 12 | 2570 | 0.13 | 0.09-0.19 | -8.50 | <.001 | 121.11 | 11 | <.001 | 90.92 |
|  | 20% method | 9 | 1728 | 0.27 | 0.19-0.36 | -4.57 | <.001 | 79.77 | 8 | <.001 | 89.97 |
|  | RCI method | 1 | 44 | 0.27 | 0.16-0.42 | -2.93 | .003 | <.001 | 0 | 1.00 | 0.00 |
|  | Cutoff method | 3 | 268 | 0.08 | 0.02-0.29 | -3.08 | .002 | 13.32 | 2 | .001 | 84.98 |
| Post 6-months to 1-year | Averaged over all CI classifications | 11 | 2939 | 0.25 | 0.17-0.34 | -4.67 | <.001 | 241.70 | 10 | <.001 | 95.86 |
|  | 1 SD method | 4 | 2080 | 0.22 | 0.11-0.40 | -2.88 | .004 | 145.08 | 3 | <.001 | 97.93 |
|  | 20% method | 6 | 689 | 0.28 | 0.15-0.45 | -2.47 | .014 | 81.83 | 5 | <.001 | 93.89 |
|  | RCI method | 2 | 342 | 0.19 | 0.07-0.41 | -2.66 | .008 | 13.01 | 1 | <.001 | 92.31 |
|  | Cutoff method | - | - | - | - | - | - | - | - | - | - |
| Post 1-year to 3-years | Averaged over all CI classifications | 4 | 203 | 0.38 | 0.27-0.51 | -1.89 | .059 | 8.03 | 3 | .045 | 62.63 |
|  | 1 SD method | 1 | 88 | 0.50 | 0.40-0.60 | 0.00 | 1 | 0.00 | 0 | 1 | 0.00 |
|  | 20% method | 1 | 35 | 0.43 | 0.28-0.59 | -0.84 | .400 | 0.00 | 0 | 1.00 | 0.00 |
|  | RCI method | 1 | 48 | 0.27 | 0.16-0.41 | -3.06 | .002 | <.001 | 0 | 1.00 | 0.00 |
|  | Cutoff method | - | - | - | - | - | - | - | - | - | - |
| Post 3-years to 5-years | Averaged over all CI classifications | 5 | 649 | 0.39 | 0.32- 0.46 | -2.91 | .004 | 10.70 | 4 | .030 | 62.61 |
|  | 1 SD method | 3 | 355 | 0.37 | 0.24- 0.53 | -1.57 | .117 | 9.34 | 2 | .009 | 78.58 |
|  | 20% method | 2 | 294 | 0.41 | 0.23- 0.61 | -0.87 | .384 | 7.11 | 1 | .008 | 85.93 |
|  | RCI method | 1 | 240 | 0.34 | 0.28-0.40 | -4.83 | <.001 | <.001 | 0 | 1.00 | 0.00 |
|  | Cutoff method | - | - | - | - | - | - | - | - | - | - |
| Post 5-years | Averaged over all CI classifications | 2 | 285 | 0.16 | 0.03-0.57 | -1.69 | .092 | 19.60 | 1 | <.001 | 94.90 |
|  | 1 SD method | - | - | - | - | - | - | - | - | - | - |
|  | 20% method | - | - | - | - | - | - | - | - | - | - |
|  | RCI method | 1 | 189 | 0.33 | 0.26-0.40 | -4.63 | <.001 | 0.00 | 0 | 1.00 | 0.00 |
|  | Cutoff method | 1 | 96 | 0.06 | 0.03-0.13 | -6.43 | <.001 | 0.00 | 0 | 1.00 | 0.00 |

^†^ All studies based on cognitive test data of control subjects, published norms or relevant population means.

^††^ Millar (2001) based on age related norms.

Supplementary Table 3

*Study specific information regarding instruments utilised and method of classification/diagnosis utilised*

| Reference No. | Lead Author, Year | Instruments | Definitions of Cognitive Impairment, Dementia and Delirium (Standardised Delirium Measurement Tool: Y/N) |
| --- | --- | --- | --- |
| [1] | Ahlgren, 2003 | Cognition: On Paper -trails A & B, Rey CFT, RAVLT (total learning, early recall, delayed recall, recognition). Computerised - K-test, simple reaction time, two choice visual stimuli RT, complex RT, simultaneous capacity | Cognition: 1 SD method (<20% tests) |
| [2] | Al Tmimi, 2016 | Delirium: CAM or CAM-ICU | Delirium: Positive CAM/CAM-ICU score (Y) |
| [3] | Alex, 2005 | Cognition: Trails A & B, grooved pegboard, RAVLT, adult memory and information-processing table A, digit span (forward & backward) | Cognition: 1 SD method |
| [4] | Anastasiadis, 2011 | Cognition: JLO, stroop test, symbol digit modalities test, digit span (forward & backward), FOME | Cognition: 1 SD method |
| [5] | Andrew, 2001 | Cognition: NART-R (at baseline), CVLT, grooved pegboard (PegR, PegL, PegRL), COWAT, trails A & B, WAIS-R digit symbol, BNT | Cognition: RCI method |
| [6] | Arrowsmith, 1998 | Cognition: RAVLT, non-verbal recognition memory, trails A & B, WAIS block design, tapping test, letter cancellation, symbol digit replacement, choice RT, displaced RT | Cognition: 1 SD method |
| [7] | Aykut, 2013 | Cognition: MoCA | Cognition: Cutoff method |
| [8] | Baba, 2007 | Cognition: HDS, Kana pick-out test, digit symbol, digit span (forward & backward) | Cognition: 20% method |
| [9] | Bassano, 2016 | Delirium: DSM criteria | Delirium: DSM criteria and requiring prolonged mechanical ventilation and/or ICU stay (N) |
| [10] | Bi, 2014 | Cognition: MMSE, CDR, GDS | Cognition: Miscellaneous |
| [11] | Bily, 2015 | Delirium: CAM-ICU, RASS | Delirium: Positive CAM-ICU score (4-step algorithm) (Y) |
| [12] | Bonacchi, 2006 | Delirium: DSS | Delirium: Patient met criteria specific to study (N) |
| [13] | Boodhwani, 2006 | Cognition: Total learning free recall, consistent long-term retrieval, long-term retrieval, long-term storage, delayed recall, digit span (forward & backward), trails A & B, grooved pegboard, symbol digit modalities, RAVLT, Buschke selective reminding, WMS-III/mental control | Cognition: 0.5 SD method |
| [14] | Borger, 2001 | Cognition: RAVLT, RVDLT, trails A & B, grooved pegboard, WMS mental control, WMS-R digit span, WMS-R visual span, verbal fluency test, AMNART | Cognition: 20% method |
| [15] | Borger, 2002 | Cognition: RAVLT, trails A and B, grooved pegboard, AMNART | Cognition: 20% method |
| [16] | Braekken, 1998 | Cognition: WAIS vocabulary, WAIS picture completion, RCPM, CVLT-L, CVLT-S, CVLT-L, serial digit learning, WMS drawing, trails A & B, letter cancellation task, WAIS digit symbol, computerised RT, COWAT, grooved pegboard | Cognition: 1 SD method |
| [17] | Breu, 2015 | Delirium: ICDSC, BPS, RASS (ICDSC only conducted when BPS was below 5) | Delirium: ICDSC score ≥ 4 (Y) |
| [18] | Breuer, 1983 | Delirium: MSE | Delirium: MSE criteria. Formal psychological test batteries were not employed (N) |
| [19] | Brown, 2016 | Delirium: CAM and MMSE, Digit Span (Forwards and Backwards), timed months of the year backwards (for non-intubated patients) or CAM-ICU (for intubated patients), DRS-R-98 (for severity) | Delirium: Positive CAM/CAM-ICU score (Y) |
| [20] | Bruce, 2013 | Cognition: COWAT, grooved pegboard (dom & non-dom), MCG complex figures, RAVLT-L, RAVLT-R, SCIT | Cognition: RCI method |
| [21] | Bucerius, 2004 | Delirium: APA guidelines | Delirium: Diagnosis made by physicians involved in care of patients according to APA guidelines (N) |
| [22] | Bucerius, 2005 | Delirium: APA guidelines | Delirium: According to APA guidelines (N) |
| [23] | Butterworth, 1999 | Cognition: Trails A & B, grooved pegboard (dom & non-dom), finger tapping (dom & non-dom), digit symbol, letter cancellation, nonverbal memory test, visual RT | Cognition: 20% method |
| [24] | Butterworth, 2005 | Cognition: "The standard neuropsychologic battery" | Cognition: 20% method |
| [25] | Buziashvili, 2005 | Cognition: MMSE, FAB, Schult test, memory (immediate and delayed reproduction of auditory & visual material) | Cognition: RCI method |
| [26] | Chakravarthy, 2008 | Cognition: MMSE | Cognition: Cutoff method |
| [27] | Chernov, 2006 | Cognition: RAVLT, digit span (forward & backward), token test, digit symbol, DCT, trails A & B, CFT | Cognition: 20% method |
| [28] | Christiansen, 2016 | Cognition: VVLT, CST, SCWT, LDCT | Cognition: 20% method |
| [29] | Colak, 2015 | Cognition: MMSE, colour trail test, grooved pegboard | Cognition: Miscellaneous |
|  |  | Delirium: DSS | Delirium: Patient met criteria specific to study (N) |
| [30] | Cook, 2007 | Cognition: RAVLT, non-verbal memory test, symbol-digit modalities, Letter cancellation, trails A & B, grooved pegboard (dom & non-dom), finger tapping test (dom & non-dom) | Cognition: 20% method |
| [31] | Cumurcu, 2008 | Delirium: DRS (for severity), DSM-IV-TR criteria, MMSE | Delirium: DSM-IV-TR diagnostic criteria (N) |
| [32] | Dabrowski, 2010 | Cognition: MMSE | Cognition: Cutoff method |
| [33] | de Tournay-Jette, 2011 | Cognition: MMSE (pre-screen, excluded if <24 pre-surgery), logical memory subtest (of the Rivermead battery), RAVLT, digit symbol, trails A & B, stroop test, verbal fluency test | Cognition: 1 SD method |
| [34] | de Baar, 2003 | Cognition: RAVLT-L, RAVLT-R, grooved pegboard, trails A & B, Sternberg memory comparison, line orientation test, stroop test | Cognition: 20% method |
| [35] | de Tournay-Jette, 2012 | Cognition: MMSE (pre-screen, excluded if <24 pre-surgery), logical memory subtest (of the Rivermead battery), RAVLT, digit symbol, trails A & B, stroop test, verbal fluency test | Cognition: 1 SD method |
| [36] | Dieleman, 2009 | Cognition: RAVLT-L, RAVLT-R, grooved pegboard, trails A & B, Sternberg memory comparison, line orientation test, stroop test, continuous performance task, self-ordering tasks, visuospatial working memory, symbol digit modalities | Cognition: RCI method |
| [37] | Djaiani, 2007 | Cognition: RAVLT, RVDLT, trails A & B, grooved pegboard, WMS digit span (forward & backward), WMS spatial span (forward & backward), choice RT, simple RT, verbal fluency test. | Cognition: Z-score method |
| [38] | Djaiani, 2008 | Delirium: NEECHAM confusion scale | Delirium: NEECHAM score ≤ 24 (Y) |
| [39] | Djaiani, 2012 | Cognition: RAVLT, RVDLT, trails A & B, grooved pegboard, WMS digit span (forward & backward), WMS spatial span (forward & backward), choice RT, simple RT, verbal fluency test. | Cognition: Z-score method |
| [40] | Dong, 2014 | Cognition: 12 neuropsychological tests used to assess cognitive functions including attention, memory and executive function | Cognition: RCI method |
|  |  | Delirium: CAM-ICU | Delirium: Positive CAM-ICU score (Features 1 and 2 are present and either Feature 3 or 4 is present) (Y) |
| [41] | Dowd, 2001 | Cognition: MMSE, trails A, HVLT (total recall, delayed free recall & discriminability index) | Cognition: 20% method |
| [42] | Dumas, 1999 | Cognition: RT, finger tapping, digit symbol, COWAT, trails A & B, digit span, Bells test, verbal nonverbal cancellation tasks. Rivermead stories, SCL-90-R (only administered preoperatively) | Cognition: 20% method |
| [43] | Ebert, 2001 | Cognition: MMSE-O, MMSE-C, COWAT, naming (of 10 line drawings of common objects), arithmetic (12 tasks involving simple operations), verbal memory (10 words with 4 free recall learning trials), visuoperceptive abilities (clock reading task) | Cognition: 1 SD method |
|  |  | Delirium: DSM-III-R | Delirium: DSM-III-R diagnostic criteria (N) |
| [44] | Eriksson, 2002 | Delirium: CAM and OBS scale | Delirium: Positive CAM score and fullfilled DSM-IV criteria (Y) |
| [45] | Eryomina, 2015 | Cognition: MMSE, FAB, clock drawing test, Schulte's tables, audio-verbal and visual memory tasks, memorising words, visualizing memorizing of a word list, verbal fluency, digit span | Cognition: Miscellaneous (pre-surgery), 20% method (post-surgery) |
| [46] | Evered, 2009 | Cognition: CERAD-AVLT, digit symbol, trails A & B, COWAT, semantic fluency, grooved pegboard (dom & non-dom) | Cognition: 1 SD method |
| [47] | Evered, 2010 | Cognition: CERAD-AVLT, digit symbol substitution, trails A & B, COWAT, CERAD semantic fluency, grooved pegboard (dom & non-dom) | Cognition: 1 SD method |
| [48] | Evered, 2011 | Cognition: CERAD-AVLT, digit symbol substitution, trails A & B, COWAT, CERAD semantic fluency, grooved pegboard (dom & non-dom) | Cognition: RCI method |
| [49] | Evered, 2016 | Cognition: CERAD-AVLT, digit symbol substitution, trails A & B, COWAT, CERAD semantic fluency, grooved pegboard (dom & non-dom) | Cognition: 1 SD method (pre-surgery), RCI method (post-surgery) |
|  |  | Dementia: CDR, informant questionnaire for cognitive decline in the elderly, CERAD-AVLT recall component, MMSE, IADLQ, GDS | Dementia: Diagnosis by academic old-age psychiatrist, informed by measures |
| [50] | Farhoudi, 2010 | Cognition: MMSE | Cognition: Cutoff method |
| [51] | Forrest, 2011 | Cognition: AVLT, stroop test, trails A & B, grooved pegboard (dom & non-dom) | Cognition: RCI method |
| [52] | Gasparovic, 2013 | Delirium: STS | Delirium: in accordance with STS definition (N) |
| [53] | Ghafari, 2012 | Cognition: MMSE | Cognition: Cutoff method |
| [54] | Goto, 2000 | Cognition: HDS | Cognition: Cutoff method |
| [55] | Goto, 2001 | Cognition: HDS | Cognition: Cutoff method |
| [56] | Goto, 2003 | Cognition: HDS | Cognition: Cutoff method |
| [57] | Grigore, 2002 | Cognition: Digit symbol, digit span (forward & backward), trails B, figural memory (immediate & delayed), Randt gist (immediate, immediate verbatim, delayed & delayed verbatim) | Cognition: 1 SD method (domain) |
| [58] | Grocott, 2002 | Cognition: WAIS-R digit symbol, WAIS-R digit span (forward & backward), trails B, WMS modified visual reproduction test (immediate & delayed), Randt short story (immediate & delayed) | Cognition: 1 SD method (domain) |
| [59] | Gunaydin, 1998 | Delirium: MMSE | Delirium: Lower than the MMSE standards (N) |
| [60] | Habib, 2014 | Cognition: McNair's and Kahn Auto-evaluation, MMSE | Cognition: Cutoff method |
| [61] | Haljan, 2009 | Cognition: WAIS-III vocabulary subset, HVLT-R, BVMT-R, WAIS-III digit symbol substitution, trails A & B, WAIS-III digit span (forwards & backwards), WAIS-III spatial span (forward & backward), grooved pegboard, FAS oral verbal fluency test | Cognition: 20% method |
| [62] | Hall, 1999 | Cognition: Trails A & B, digit span (forward & backward), COWAT | Cognition: Z-score method |
| [63] | Hall, 2005 | Cognition: Verbal fluency, trails B, Pre-op digit symbol and post-op symbol digit modalities, RAVLT, ROCF test (pre-op) and TCF test (post-op) | Cognition: 1 SD method |
| [64] | Hammon, 2006 | Cognition: 11-part neuropsychological exam reported in statement of consensus (Murkin et al., 1995) | Cognition: 20% method (<20% tests) |
| [65] | Harmon, 2004 | Cognition: RAVLT, trails A & B, grooved pegboard, COWAT, digit symbol | Cognition: RCI method |
|  |  | Delirium: DSM-III-R, MMSE | Delirium: Diagnosis based on the DSM-III-R criteria and the MMSE (N) |
| [66] | Harmon, 2005 | Cognition: RAVLT, trails A & B, grooved pegboard, COWAT, digit symbol | Cognition: RCI method |
|  |  | Delirium: DSM-III-R, MMSE | Delirium: Diagnosis based on the DSM-III-R criteria and the MMSE (N) |
| [67] | Harrison, 1989 | Cognition: WAIS block design subtest, RAVLT, trails A & B, grooved pegboard, letter cancellation. Computer-based: symbol digit replacement, two-choice RT, two forms of a nonverbal memory test | Cognition: 1 SD method |
| [68] | Hernandez, 2007 | Cognition: Trails A & B, digit span, VIGIL, grooved pegboard, ROCF, COWAT, HVLT, oral reading test, WRAT-3, Brixton spatial anticipation test | Cognition: 20% method |
| [69] | Herrmann, 2000 | Delirium: BPR scale, DSM-III-R, DRS (for severity) | Delirium: DSM-III-R diagnostic criteria (N) |
| [70] | Heyer, 1997 | Cognition: MMSE, trails A & B, index tapping test, grooved pegboard (dom & non-dom), Buschke selective reminding test (long term retrieval) | Cognition: 20% method, 20% method (<20% tests) |
| [71] | Ho, 2004 | Cognition: Blessed orientation-memory-concentration test (short form), behavioural dyscontrol scale, trails A | Cognition: 1 SD method, 0.5 SD method, 20% method |
| [72] | Hudetz, 2011 | Cognition: Story memory subtest, word list memory subtest, brief visual memory test revised, digit span (backward), semantic fluency, phonemic fluency, stroop test | Cognition: Z-score method |
|  |  | Delirium: ICDSC | Delirium: ICDSC score ≥ 4 (Y) |
| [73] | Humphreys, 2016 | Delirium: DSI, SPMSQ | Delirium: Positive DSI score (had any one of the critical symptoms of delirium: disorientation, disturbance of consciousness, or perceptual disturbance) (Y) |
| [74] | Ito, 2012 | Cognition: HDS | Cognition: Cutoff method |
| [75] | Jensen, 2006 | Cognition: MMSE (for screening), VVLT, CST, stroop test, LDCT | Cognition: Miscellaneous, 20% method, RCI method |
| [76] | Jensen, 2008 | Cognition: MMSE (for screening), VVLT, CST, stroop test, LDCT | Cognition: Miscellaneous, 20% method, RCI method |
| [77] | Jonsson, 2004 | Cognition: Mental control , figural memory, logical memory (A/B), visual reproduction, RAVLT, trails A & B, digit symbol, digit span, visual memory span, visual paired associates II or verbal paired associates I, delayed retention | Cognition: 1 SD method, 20% method |
| [78] | Joung, 2013 | Cognition: SVLT, digit span (forward & backward), trails A & B, digit symbol | Cognition: 20% method |
| [79] | Kadoi, 2001 | Cognition: MMSE, RAVLT, trails A & B, digit span (forward), grooved pegboard | Cognition: 1 SD method |
| [80] | Kadoi, 2002 | Cognition: MMSE, RAVLT, trails A & B, digit span (forward), grooved pegboard | Cognition: 1 SD method |
| [81] | Kadoi, 2003 | Cognition: MMSE, RAVLT, trails A & B, digit span (forward), grooved pegboard | Cognition: 1 SD method |
| [82] | Kadoi, 2005 | Cognition: MMSE, RAVLT, trails A & B, digit span (forward), grooved pegboard | Cognition: 1 SD method |
| [83] | Kadoi, 2007 | Cognition: MMSE, RAVLT, trails A & B, digit span (forward), grooved pegboard | Cognition: 1 SD method |
| [84] | Kadoi, 2011 (a) | Cognition: MMSE, RAVLT, trails A & B, digit span (forward), grooved pegboard | Cognition: 1 SD method |
| [85] | Kadoi, 2011 (b) | Cognition: MMSE, RAVLT, trails A & B, digit span (forward), grooved pegboard | Cognition: 1 SD method |
| [86] | Kadoi, 2013 | Cognition: MMSE, RAVLT, trails A & B, digit span (forward), grooved pegboard | Cognition: 1 SD method |
| [87] | Kara, 2015 | Cognition: MoCA | Cognition: Cutoff method |
| [88] | Kaukinen, 2000 | Cognition: WAIS similarities, WAIS digit span (forward & backward), WAIS digit symbol , trails A & B, memory tests modified to Finnish | Cognition: 20% method |
| [89] | Kazmierski, 2014 (a) | Cognition: MoCA, trails B | Cognition: Cutoff method |
|  |  | Delirium: CAM or CAM-ICU, RASS | Delirium: If RASS was above –4 (–3 through +4), assessment with the CAM-ICU was administered (Y) |
| [90] | Kazmierski, 2014 (b) | Delirium: CAM-ICU, MDAS (for severity) | Delirium: Positive CAM-ICU score (Feature 1 and Feature 2 and either Feature 3 or 4 are present) (Y) |
| [91] | Keizer, 2005 | Cognition: RAVLT, Sternberg letter cancellation, trails A & B, grooved pegboard, stroop test, symbol digit modalities, subjective ordering tasks | Cognition: 1 SD method, 20% method, RCI method |
| [92] | Kempfert, 2008 | Delirium: APA guidelines | Delirium: Diagnosed by physician in accordance with APA guidelines (N) |
| [93] | Khan, 2014 | Delirium: DSM-IV | Delirium: Diagnosed using DSM-IV criteria (N) |
| [94] | Khatri, 1999 | Cognition: Randt short story, WAIS-R digit span, WAIS-R digit symbol, trails B, figural memory | Cognition: 20% method |
| [95] | Knipp, 2008 | Cognition: Trails A & B, Zimmermann joint attention test, verbal learning test (immediate recall & delayed recognition), digit span (forward and backward), Corsi block tapping test (forward & backward), Horn performance test 55+ (subtests 3 & 9) | Cognition: Z-score method |
| [96] | Kok, 2014 | Cognition: CogState brief computerised test battery (detection task, identification task, one card learning task and one back task) | Cognition: RCI method |
| [97] | Kok, 2017 | Cognition: CogState brief computerised test battery (detection task, identification task, one card learning task and one back task) | Cognition: RCI method |
| [98] | Kozora, 2010 | Cognition: WMS-III logical memory subtest, WMS-III faces subtest, WAIS-III digit span, WAIS-III digit symbol, trails A & B, clock drawing | Cognition: 1 SD method |
| [99] | Krzych, 2014 | Delirium: DSM-IV, physician diagnosis | Delirium: Diagnosed predominantly by attending physician according to DSM-IV criteria (N) |
| [100] | Lahariya, 2014 | Delirium: CAM-ICU, DSM-IV-TR, RASS | Delirium: CAM-ICU, DSM-IV-TR diagnostic criteria by psychiatrist (Y) |
| [101] | Leacche, 2003 | Delirium: DSM-III-R | Delirium: Diagnosed by psychiatrist in accordance with DSM-III-R criteria (N) |
| [102] | Lee, 2003 | Cognition: WAIS-III vocabulary subtest, RAVLT, Benton visual retention test, trails A & B, grooved pegboard, finger tapping, WAIS-III digit symbol subtest | Cognition: 20% method |
| [103] | Lee, 2005 | Dementia: ICD-9 criteria: history, mental status exam, physical exam, neuro exam, blood chemistry | Dementia: Diagnosed with Altzheimer's Disease (ICD-9) by clinical criteria common to community practitioners - history from patient, relative or friend, mental status exam (e.g.,MMSE), physical exam, neurological exam, blood chemistry |
| [104] | Lelis, 2006 | Cognition: MMSE | Cognition: Cutoff method |
| [105] | Lewis, 2006 (a) | Cognition: NART (at baseline), CERAD word learning task, trails A & B, digit symbol, COWAT, grooved pegboard (dom & non-dom) | Cognition: 1 SD method |
| [106] | Lewis, 2006 (b) | Cognition: NART (at baseline), CERAD word learning task, trails A & B, digit symbol, COWAT, grooved pegboard (dom & non-dom) | Cognition: 20% method, 1 SD method, RCI method |
| [107] | Lewis, 2007 | Cognition: NART (at baseline), CERAD word learning task, trails A & B, digit symbol, COWAT, grooved pegboard (dom & non-dom) | Cognition: RCI method |
| [108] | Li, 2015 | Delirium: CAM | Delirium: Positive CAM score (Features 1 and 2 are present and either Feature 3 or 4 is present) (Y) |
| [109] | Liu, 2009 | Cognition: WMS mental control, WMS visual retention, WMS paired-associate verbal learning, digit span (forward and backward), WAIS-R digit symbol, trails A, grooved pegboard (dom & non-dom) | Cognition: RCI method |
| [110] | Loponen, 2008 | Delirium: DSS (clinically diagnosed) | Delirium: Clinically diagnosed with requirement that temporary medication, i.e. diazepam or haloperidol, was needed to sedate the delirious patient (N) |
| [111] | Lund, 2003 | Cognition: Grooved pegboard (dom & non-dom), digit symbol, trails A & B, digit span (forward & backward), stroop test, RAVLT, WAIS-R similarities and vocabulary, COWAT, WAIS-R picture completion and block design | Cognition: 20% method (<20% tests) |
| [112] | Lund, 2005 | Cognition: Grooved pegboard, WAIS-R digit symbol, trails A & B, WAIS-R digit span, stroop test, RAVLT, WAIS-R similarities, COWAT, WAIS-R block design | Cognition: 20% method |
| [113] | Maekawa, 2011 | Cognition: HDS, digit span (forward & backward), digit symbol, Kana pick-out test | Cognition: Cutoff method (for baseline), 20% method (for follow-up) |
| [114] | Mahanna, 1996 | Cognition: Randt short story (immediate & delayed recall), WAIS digit symbol, digit span (forward & backward), trails B, Benton revised visual retention test | Cognition: 1 SD method, 20% method, Miscellaneous |
| [115] | Mardani, 2012 | Delirium: DSM-IV, MMSE | Delirium: DSM-IV criteria interviews conducted on patients with a MMSE score ≤ 23 (N) |
| [116] | Mariscalco, 2012 | Delirium: CAM-ICU | Delirium: At least 2 positive assessments on CAM-ICU (Features 1 and 2 are present and either Feature 3 or 4 is present) (Y) |
| [117] | Martens, 2004 | Cognition: Block design, Benton revised retention test, TMT, digit span, d2 test of attention | Cognition: 20% method |
| [118] | Martin, 2010 | Delirium: STS | Delirium: Defined according to STS definition (N) |
| [119] | Martin, 2012 | Delirium: STS | Delirium: Defined according to STS definition (N) |
| [120] | Mathew, 2003 | Cognition: Randt short story, WMS figural memory test, WAIS-R digit symbol subtest, trails B, RAVLT, WAIS-R digit span | Cognition: 1 SD method (domain) |
| [121] | Mathew, 2005 | Cognition: Randt short story, WMS modified visual reproduction test, WAIS-R digit span, WAIS-R digit symbol, trails B | Cognition: 1 SD method (domain) |
| [122] | Mathew, 2006 | Cognition: Randt short story, WMS modified visual reproduction test, WAIS-R digit span, WAIS-R digit symbol, trails B | Cognition: 1 SD method (domain) |
| [123] | Mathew, 2007 | Cognition: Randt short story, WMS modified visual reproduction test, WAIS-R digit span, WAIS-R digit symbol, trails B | Cognition: 1 SD method (domain) |
| [124] | Millar, 2001 | Cognition: Stroop test | Cognition: Cutoff method |
| [125] | Miyazaki, 2011 | Delirium: DSM-IV | Delirium: Diagnosed according to DSM-IV criteria or administering antipsychotic agents by reviewing medical records during the ICU stay (N) |
| [126] | Molstrom, 2016 | Cognition: MMSE | Cognition: Cutoff method |
| [127] | Mongero, 2001 | Cognition: MMSE, TMT, repetitive tapping test, Buschke verbal selective reminding test, grooved pegboard | Cognition: 20% method |
| [128] | Mu, 2010 | Delirium: CAM-ICU, RASS | Delirium: If RASS was above –4 (–3 through +4), assessment with the CAM-ICU was administered (4-step algorithm) (Y) |
| [129] | Mu, 2013 | Cognition: Trails A, grooved pegboard (dom & non-dom), the WMS-Chinese edn. of the mental control subtest, digit span subtest (forward & backward), visual retention subtest, paired associate verbal learning subtest, digit symbol subtest | Cognition: 1 SD method (preop) / RCI method |
| [130] | Mullges, 2002 | Cognition: Trails A & B, d2-letter cancellation, Bentons visual retention test, WAIS block design test, WAIS digit span | Cognition: 1 SD method |
| [131] | Murkin, 1995 | Cognition: WMS digit span, WMS mental control, WAIS-R digit symbol, grooved pegboard, WMS verbal paired associates | Cognition: Miscellaneous |
| [132] | Mutch, 2011 | Dementia: ICD-9-CM criteria: hospital abstract data/physician visits | Dementia: Defined from hospital abstract data/physician visits, ICD-9-CM code diagnoses |
| [133] | Nathan, 2007 | Cognition: Buschke total learning free recall, Buschke consistent long-term retrieval, Buschke long-term retrieval, Buschke long-term storage, Buschke delayed recall, digit span (forwards & backwards), trails A & B, grooved pegboard (dom), symbol digit modalities | Cognition: 1 SD method (domain) |
| [134] | Newman, 2001 | Cognition: Randt short story, WAIS-R digit span (forward & backward), Benton revised visual retention test, WAIS-R digit symbol, trails B | Cognition: 1 SD method (domain) |
| [135] | Nikolic, 2012 | Delirium: CAM | Delirium: Positive CAM score (Features 1 and 2 are present and either Feature 3 or 4 is present) (Y) |
| [136] | Norkiene, 2007 | Delirium: DSM-IV | Delirium: Clinician diagnosis according to DSM-IV criteria (N) |
| [137] | Norkiene, 2011 | Cognition: MMSE, RAVLT, trails A & B, digit span, digit symbol, cube drawing | Cognition: 1 SD method |
|  |  | Delirium: DSM-IV | Delirium: Defined according to DSM-IV criteria (N) |
| [138] | Oh, 2008 | Cognition: MMSE, trails A, grooved pegboard | Cognition: 20% method |
| [139] | Oldham, 2015 | Cognition: CDR, MMSE, digit span, HVLT, WMS-IV, progressive digit sequencing, three word fluency tasks, NAB mazes subtest, trails A & B, digit symbol | Cognition: Cutoff method, 1 SD method |
|  |  | Delirium: aDST, CAM, DI, MMSE | Delirium: Determined based on CAM (Y) |
| [140] | Omiya, 2015 | Delirium: DRS-R-98 | Delirium: DRS-R-98 score ≥ 8 (Y) |
| [141] | Osse, 2012 | Delirium: CAM-ICU | Delirium: Positive CAM-ICU score lasting 2 days or longer (Y) |
| [142] | Otomo, 2013 | Delirium: DSM-IV, DRS | Delirium: Diagnosed according to DSM-IV criteria & DRS score (Y) |
| [143] | Ozturk, 2016 | Cognition: MMSE | Cognition: Miscellaneous |
| [144] | Palmbergen, 2012 | Delirium: DOS scale, confirmed by geriatrician or internist | Delirium: DOS scale for screening. If suspected, confirmed by geriatrician or internist (Y) |
| [145] | Patel, 1996 | Cognition: RAVLT, computer administrated checkerboard test, trails A & B, letter cancellation task, choice RT, symbol digit replacement, grooved pegboard (dom & non-dom), WAIS block design | Cognition: 1 SD method |
| [146] | Peterson, 2012 | Cognition: WAIS-R digit span, trails A & B, Boston naming, Benton visual retention test, COWAT, WAIS-R digit symbol, Mattis-Kovner verbal recall and recognition, finger tapping test, Ammons quick test (used as proxy for verbal IQ) | Cognition: Miscellaneous |
| [147] | Phillips-Bute, 2006 | Cognition: Randt short story, WAIS-R digit span, WMS modified visual reproduction, WAIS-R digit symbol, trails B | Cognition: 1 SD method (domain) |
| [148] | Prakanrattana, 2007 | Delirium: CAM-ICU | Delirium: Positive CAM-ICU score (Features 1 and 2 are present and either Feature 3 or 4 is present) (Y) |
| [149] | Ramlawi, 2006 | Cognition: HVLT, BNT, trails A & B, WAIS-R digit span (forward & backward), fluency tasks (semantic & phonemic), Wechsler test of adult reading, stroop test | Cognition: 1 SD method |
| [150] | Rasmussen, 1999 | Cognition: MMSE (for screening), VVLT, CST, stroop test, LDCT | Cognition: RCI method |
| [151] | Rasmussen, 2002 | Cognition: MMSE (for screening), VVLT, CST, stroop test, LDCT | Cognition: RCI method |
| [152] | Raymond, 2006 | Cognition: MicroCog - Assessment of Cognitive Functioning (attention/mental control, reasoning/calculation, memory, spatial processing, RT, information processing (speed & accuracy), general cognitive (functioning & proficiency) | Cognition: Miscellaneous (standardised regression-based technique), RCI method (using 1.645 z-score), 1 SD method, 20% method |
| [153] | Reents, 2002 | Cognition: d2-letter cancellation test, trails B, Benton‘s visual retention test, WAIS block design, WAIS digit span | Cognition: 1 SD method |
|  |  | Delirium: DSM-IV | Delirium: Defined according to DSM-IV criteria (N) |
| [154] | Restrepo, 2002 | Cognition: Trails B, oral and written naming test, oral reading tests, line cancellation, Bells tests | Cognition: Z-score method |
| [155] | Ringaitiene, 2015 | Delirium: CAM-ICU | Delirium: Positive CAM-ICU score (Y) |
| [156] | Robson, 2000 | Cognition: RAVLT, trails A & B, PASAT, grooved pegboard, COWAT, NART, block design, object assembly test, digit symbol, picture completion test | Cognition: 1 SD method (<20% tests), 0.5 SD method (<20% tests) |
| [157] | Rolfson, 1999 | Delirium: DSM-III-R on clinical grounds (CAM, CAM-MD, CAM-RN, MMSE, clock drawing test, MD chart review, RN chart review - used to determine clinical diagnosis) | Delirium: Clinically diagnosed according to DSM-III-R criteria, based on results from standardised measures (Y) |
| [158] | Royse, 2000 | Cognition: Recall (short-term & delayed), COWAT, trails A & B, grooved pegboard (dom & non-dom), digit symbol, digit span (forward & backward) | Cognition: 20% method |
| [159] | Royse, 2011 | Cognition: Trails A & B, COWAT, stroop test, letter cancellation, grooved pegboard (dom & non-dom), RAVLT, digit span (forward & backward), symbol digit modalities | Cognition: 1 SD method (<20% tests) |
|  |  | Delirium: CAM | Delirium: Positive CAM score (Y) |
| [160] | Rudolph, 2005 | Delirium: CAM, digit span, DSI, MDAS, MMSE | Delirium: Positive CAM score (Features 1 and 2 are present and either Feature 3 or 4 is present) (Y) |
| [161] | Rudolph, 2006 | Delirium: CAM (CAM-ICU for postoperatively intubated patients), digit span, DSI, MDAS, MMSE | Delirium: Positive CAM score (Features 1 and 2 are present and either Feature 3 or 4 is present) (Y) |
| [162] | Rudolph, 2009 | Delirium: CAM, digit span, DSI, MDAS, MMSE | Delirium: Positive CAM score (Features 1 and 2 are present and either Feature 3 or 4 is present) (Y) |
| [163] | Saczynski, 2012 | Delirium: CAM, digit span test, DSI, MMSE | Delirium: Positive CAM score (Features 1 and 2 are present and either Feature 3 or 4 is present) (Y) |
| [164] | Santos, 2005 | Delirium: DSM-IV | Delirium: Diagnosed according to DSM-IV criteria (N) |
| [165] | Scott, 2002 | Cognition: WMS-R logical memory (I & II), altered form of WMS-R digit span, trails A & B, COWAT | Cognition: 1 SD method (<20% tests), 1 SD method |
| [166] | Sellman, 1993 | Cognition: Synonyms, reasoning, block design, Halstead category test, tactual performance test (dom, non-dom, memory, localization), trails A & B, Claeson Dahl, memory-for-designs test | Cognition: 1 SD method, 1 SD method (<20% tests) |
| [167] | Selnes, 2008 | Cognition: MMSE | Cognition: Cutoff method |
| [168] | Sevuk, 2015 | Delirium: DRS-R-98 (for severity), ICDSC | Delirium: ICDSC score ≥ 4 (Y) |
| [169] | Shaw, 1986 | Cognition: Trails B, WMS (information, orientation, mental control, logical memory, digits total, visual reproduction, associate learning subtests), WAIS block design, WAIS vocabulary | Cognition: 1 SD method (<20% tests), 1 SD method |
| [170] | Shaw, 1987 | Cognition: Trails B, WMS (information, orientation, mental control, logical memory, digits total, visual reproduction, associate learning subtests), WAIS block design, WAIS vocabulary | Cognition: 1 SD method (<20% tests), 1 SD method |
| [171] | Shioiri, 2016 | Delirium: DRS (for severity), DSM-IV-TR | Delirium: Psychiatrist diagnosis according to DSM-IV-TR criteria (N) |
| [172] | Siepe, 2011 | Delirium: MMSE, psychologist assessment | Delirium: 10 point drop or more on MMSE from pre-op and a positive assessment by a psychologist (N) |
| [173] | Silbert, 2004 | Cognition: Method 1 - CERAD word learning test, symbol digit modalities, trails A & B, semantic fluency, grooved pegboard. Method 2 - CogState battery (RT & accuracy of detection, identification & matching in playing card tasks) | Cognition: RCI method (uses 1.65 z-score) |
| [174] | Silva, 2016 | Cognition: VLT, stroop test, trails A & B, symbol digit modalities | Cognition: 20% method |
|  |  | Delirium: CAM-ICU | Delirium: Positive CAM-ICU score on at least one of two testing days (Y) |
| [175] | Sirvinskas, 2014 | Cognition: MMSE, WMS modified visual reproduction test, trails A & B, WAIS digit span, WAIS digit symbol substitution | Cognition: 1 SD method (domain) |
| [176] | Slater, 2009 | Cognition: MMSE, Trails A & B, HVLT (trials 1, 2, 3, B & C), grooved pegboard (dom & non-dom), stroop test (part C & CW) | Cognition: 1 SD method (<20% tests) |
|  |  | Delirium: DRS | Delirium: Based on DRS (Y) |
| [177] | Smith, 1986 | Cognition: WAIS vocab and picture completion subtests, RAVLT, block design, grooved pegboard, trails A & B, letter cancellation, digit symbol replacement, two-choice RT | Cognition: 1 SD method |
| [178] | Smith, 1988 | Cognition: RAVLT, non-verbal memory test, grooved pegboard, symbol digit replacement, letter cancellation, choice RT, block design, WAIS vocab and picture completion (preop only) | Cognition: 1 SD method |
| [179] | Soehle, 2015 | Delirium: CAM-ICU, RASS | Delirium: Positive CAM-ICU score (Y) |
| [180] | Song, 2016 | Cognition: MMSE, MoCA | Cognition: Cutoff method |
| [181] | Stanley, 2002 | Cognition: Randt short story, WAIS-R digit span, WMS figural memory, WAIS-R digit symbol, trails B, RAVLT | Cognition: 1 SD method (domain) |
| [182] | Stroobant, 2002 | Cognition: RAVLT, trails B, grooved pegboard, TAPS, LBT, COWAT, JLO | Cognition: 20% method |
| [183] | Stroobant, 2005 | Cognition: RAVLT, trails B, grooved pegboard, TAPS, LBT, COWAT, JLO | Cognition: 20% method, 20% method (<20% tests) |
| [184] | Stroobant, 2008 | Cognition: RAVLT, trails B, grooved pegboard, TAPS, LBT, COWAT, JLO | Cognition: 20% method |
| [185] | Suksompong, 2002 | Cognition: Thai Mental State Exam | Cognition: Miscellaneous |
| [186] | Svenmarker, 2004 | Cognition: Three tasks from SuperLab: Presentation of a set of 40 line drawing from Snodgrass's standardized collection, two explicit memory tests, one implicit memory test | Cognition: 1 SD method |
| [187] | Swaminathan, 2002 | Cognition: Randt short story (immediate & delay), digit symbol, trails B, digit span (forward & backward), figural memory (immediate & delayed) | Cognition: 1 SD method (domain) |
| [188] | Sylivris, 1998 | Cognition: WAIS-R general information questionnaire, digit span, digit symbol, RAVLT, COWAT | Cognition: Miscellaneous |
| [189] | Szwed, 2014 | Cognition: Stroop test (A & B), digit span (forward & backward), FAS version of COWAT | Cognition: 20% method |
| [190] | Tabatabaie, 2015 | Delirium: CAM-ICU | Delirium: Positive CAM-ICU score (Y) |
| [191] | Tagarakis, 2007 | Delirium: DRS | Delirium: Based on DRS, which was performed on patients suspected to develop delirium (Y) |
| [192] | Taggart, 2003 | Cognition: Standard battery of 10 cognitive tests (Murkin et al., 1995): inclusive of the RAVLT, adult memory and information processing battery, trails B, verbal fluency | Cognition: 20% method |
| [193] | Tan, 2008 | Delirium: CAM, MDAS and MMSE | Delirium: Positive CAM score (Features 1 and 2 are present and either Feature 3 or 4 is present) (Y) |
| [194] | Thornton, 2005 | Cognition: RAVLT, trails A & B, grooved pegboard (dom & non-dom) | Cognition: 20% method |
| [195] | Toeg, 2013 | Cognition: Buschke selective reminding or RAVLT, WAIS-R digit span, finger tapping task, letter and category fluency, trails A & B, grooved pegboard, symbol digit modalities | Cognition: 1 SD method (domain) |
| [196] | Toner, 1994 | Cognition: RAVLT, 2 computerised non-verbal learning tests (NVM levels 1,2), trails A & B, letter cancellation, grooved pegboard, two choice RT, digit symbol replacement, block design, WAIS vocab and picture completion test | Cognition: 1 SD method |
| [197] | Toner, 1997 | Cognition: RAVLT, 2 computerised non-verbal learning tests (NVM levels 1,2), trails A & B, letter cancellation, grooved pegboard, two choice RT, digit symbol replacement, block design, WAIS vocab and picture completion test | Cognition: 1 SD method |
| [198] | Toner, 1998 | Cognition: RAVLT, 2 computerised non-verbal learning tests (NVM levels 1,2), trails A & B, letter cancellation, grooved pegboard, two choice RT, digit symbol replacement, block design, WAIS vocab and picture completion test | Cognition: 1 SD method |
| [199] | Trubnikova, 2014 | Cognition: Complex visuomotor reaction (reaction time, number of errors), functional mobility of nervous processes and performance of brain responses to feedback (reaction time, number of errors, missed signals), Bourdons test, visual short term memory tests (memorisation of 10 numbers, 10 words, 10 nonsense syllables) | Cognition: 20% method |
| [200] | Tse, 2015 | Delirium: attending physician, CAM, DSM-IV-TR | Delirium: Screened using CAM and clinical diagnosis made by physician (Y) |
| [201] | Tully, 2010 | Delirium: DSI, DSM-IV-TR, SPMSQ | Delirium: Classification based on DSM-IV-TR criteria. Evidence of perceptual disturbance and/or language disturbance was requisite for a delirium diagnosis. Neurology assessments, SPMSQ results and medical notes also evaluated. (N) |
| [203] | van Dijk, 2002 | Cognition: RAVLT-L, RAVLT-R, grooved pegboard, trails A & B, Sternberg memory comparison, line orientation test, stroop test, continuous performance task, self-ordering tasks, visuospatial working memory, symbol digit modalities | Cognition: 20% method |
| [204] | van Dijk, 2004 | Cognition: RAVLT-L, RAVLT-R, grooved pegboard, trails A & B, Sternberg memory comparison, line orientation test, stroop test | Cognition: 20% method |
| [205] | van Dijk, 2007 | Cognition: RAVLT-L, RAVLT-R, grooved pegboard, trails A & B, Sternberg memory comparison, line orientation test, stroop test, continuous performance task, self-ordering tasks, visuospatial working memory, symbol digit modalities | Cognition: 20% method, RCI method |
| [202] | Vanninen, 1998 | Cognition: WMS logical prose subtest (story A - immediate & delayed recall), list learning test (modified from AVLT), visual reproduction, digit symbol, alternating S task, stroop test (A, B, C), reaction time, choice RT, finger tapping | Cognition: 1 SD method (<20% tests) |
| [206] | Vedin, 2006 | Cognition: Digit span (forward & backward), block span (forward/backward), trails A-D, WAIS digit symbol, COWAT-FAS, Claeson-Dahl verbal learning Test, Claeson-Dahl verbal retention | Cognition: 20% method |
| `` | Walzer, 1997 | Delirium: DSM-III-R | Delirium: Clinically diagnosed according to DSM-III-R criteria (N) |
| [208] | Wang, 2002 | Cognition: Trails A, grooved pegboard, the WMS-Chinese edn. of the mental control test, digit span (forward & backward) visual retention and paired associate verbal learning, figural memory and verbal learning/memory, measures of attention and concentration, digit symbol subtest | Cognition: 1 SD method |
| [209] | Whitaker, 2004 | Cognition: RAVLT, trails A & B, grooved pegboard (dom & non-dom), symbol digit replacement test, non-verbal memory, letter cancellation, choice RT | Cognition: 1 SD method |
| [210] | Yilmaz, 2016 | Delirium: CAM-ICU | Delirium: Positive CAM-ICU score (Features 1 and 2 are present and either Feature 3 or 4 is present) (Y) |
| [211] | Yoda, 2004 | Cognition: MMSE | Cognition: Cutoff method |
| [212] | Yoon, 2001 | Delirium: DSS | Delirium: Patient met criteria specific to study (N) |
| [213] | Zamvar, 2002 | Cognition: RAVLT, trails A & B, digit substitution test, digit span (forward & backward), grooved pegboard (dom & non-dom), COWAT | Cognition: 1 SD method |
| [214] | Zhang, 2015 | Delirium: CAM-ICU, RASS | Delirium: Positive CAM-ICU score (Features 1 and 2 are present and either Feature 3 or 4 is present) (Y) |
| [215] | Zimpfer, 2004 | Cognition: MMSE, trails A | Cognition: 1 SD method |
| Note. *see Supplementary Table 4 for index of instrument acronyms* | | | |

Supplementary Table 4

*Glossary of abbreviations*

| **Cognitive Impairment** | |
| --- | --- |
| **AMNART** | American National Adult Reading Test |
| **TAPS** | Block Taps Test |
| **BNT** | Boston Naming Test |
| **BVMT-R** | Brief Visual Retention Test |
| **CVLT** | California Verbal Learning Test |
| **CDR** | Clinical Dementia Rating scale |
| **CFT** | Complex Figure Test |
| **CST** | Concept Shifting Test |
| **COWAT** | Controlled Oral Word Association Test |
| **Digit symbol** | Digit Symbol Substitution Task |
| **FAB** | Frontal Assessment Battery |
| **FOME** | Fuld Object Memory Evaluation |
| **GDS** | Global Deterioration Scale |
| **Trails A & B** | Halstead-Reitan Trail-making tests A & B |
| **HDS** | Hasegawa Dementia Scale |
| **HVLT** | Hopkins Verbal Learning Test |
| **JLO** | Judgment of line orientation |
| **LDCT** | Letter-Digit Coding Test |
| **LBT** | Line Bisection Test |
| **MCG** | Medical College of Georgia |
| **MMSE** | Mini Mental State Examination |
| **MoCA** | Montreal Cognitive Assessment |
| **NART-R** | National Adult Reading Test–Revised |
| **NAB** | Neuropsychological Assessment Battery |
| **PASAT** | Paced Auditory Serial Addition Task |
| **Randt short story** | Randt Memory Test Short-Story Module |
| **RCPM** | Raven Coloured Progressive Matrices |
| **RT** | Reaction Time |
| **RAVLT** | Rey Auditory Verbal Learning Test |
| **RAVLT-L** | Rey Auditory-Verbal Learning – Learning Trial |
| **RAVLT-R** | Rey Auditory-Verbal Learning – Recognition Trial |
| **RVDLT** | Rey Visual Design Learning Test |
| **ROCF** | Rey-Osterrieth Complex Figure Test |
| **SVLT** | Seoul Verbal Learning Test |
| **Stroop test** | Stroop Colour Word Interference Test |
| **SCIT** | Subtle Cognitive Impairment Test |
| **TCF** | Taylor Complex Figure Test |
| **DCT** | The Bourdon-Wiersma Dot Cancellation Test |
| **MMSE-C** | The MMSE scoring for Cognitive Status |
| **MMSE-O** | The MMSE scoring for Orientation |
| **SCL-90-R** | The Symptoms Checklist-90-R |
| **AVLT** | Auditory Verbal Learning Test |
| **CERAD** | The Consortium to Establish a Registry for Alzheimer’s Disease |
| **TMT** | Trail Making Test |
| **VLT** | Verbal Learning Test |
| **VIGIL** | Vigilance Task |
| **VVLT** | Visual Verbal Learning Test |
| **WAIS** | Wechsler Adult Intelligence Scale |
| **WMS** | Wechsler Memory Scale |
| **WRAT-3** | Wide Range Achievement Test, 3^rd^ Edition |
| **Delirium** | |
| **aDST** | abbreviated Digit Span Test |
| **STS** | Accordance with Society of Thoracic Surgeons |
| **APA** | American Psychiatric Association |
| **BPS** | Behavioural Pain Scale |
| **BPR** | Brief Psychiatric Rating scale |
| **CAM** | Confusion Assessment Method |
| **CAM-ICU** | Confusion Assessment Method for the ICU |
| **DSS** | Definition that is specific to the study |
| **DI** | Delirium Index |
| **DOS** | Delirium Observation Screening scale |
| **DRS** | Delirium Rating Scale |
| **DRS-R-98** | Delirium Rating Scale Revised-98 |
| **DSI** | Delirium Symptom Interview |
| **DSM** | Diagnostic and Statistical Manual of Mental Disorders |
| **DSM-III-R** | Diagnostic and Statistical Manual of Mental Disorders 3^rd^ ed., Revised. |
| **DSM-IV** | Diagnostic and Statistical Manual of Mental Disorders 4^th^ ed. |
| **DSM-IV-TR** | Diagnostic and Statistical Manual of Mental Disorders 4^th^ ed., Text Revision |
| **MDAS** | Memorial Delirium Assessment Scale |
| **MMSE** | Mini Mental State Examination |
| **OBS** | Organic Brain Syndrome scale |
| **SPMSQ** | Short Portable Mental Status Questionnaire |
| **ICDSC** | The Intensive Care Delirium Screening Checklist |
| **MSE** | The Mental Status Examination |
| **RASS** | The Richmond Agitation Sedation Scale |
| **Dementia** | |
| **CDR** | Clinical Dementia Rating scale |
| **IADLQ** | Instrumental Activities of Daily Living Questionnaire |
| **ICD-9-CM** | International Classification of Diseases, Ninth Revision, Clinical Modification |
| **GDS** | The Geriatric Depression Scale |

Supplementary Table 5

*Pooled estimates relative to outcome (delirium and cognitive impairment), decade study was published/conducted and time point.*

|  |  | Pooled-effect (prevalence) | | | | | | Heterogeneity | | | |
| --- | --- | --- | --- | --- | --- | --- | --- | --- | --- | --- | --- |
|  |  | N studies | N participants | Point-estimate | 95%CI | Z-value | p | Q-value | df(Q) | p | I^2^ |
| Delirium | 1980s | 1 | 421 | 0.12 | 0.09-0.15 | -13.34 | <.001 | 0 | 0 | 1 | 0 |
|  | 1990s | 11 | 44484 | 0.11 | 0.09-0.14 | -16.13 | <.001 | 310.55 | 10 | <.001 | 96.78 |
|  | 2000s | 36 | 13067 | 0.16 | 0.11-0.22 | -8.28 | <.001 | 1209.37 | 35 | <.001 | 97.11 |
|  | 2010s | 22 | 3144 | 0.26 | 0.21-0.31 | -7.57 | <.001 | 185.62 | 21 | <.001 | 88.69 |
| Pre-surgery | 1980s | - | - | - | - | - | - | - | - | - | - |
|  | 1990s | - | - | - | - | - | - | - | - | - | - |
|  | 2000s | 7 | 1309 | 0.19 | 0.12-0.28 | -5.28 | <.001 | 73.17 | 6 | <.001 | 91.80 |
|  | 2010s | 6 | 945 | 0.19 | 0.10-0.33 | -3.86 | <.001 | 66.72 | 5 | <.001 | 92.51 |
| Immediate post-surgery (up to 4-days) | 1980s | - | - | - | - | - | - | - | - | - | - |
|  | 1990s | 4 | 669 | 0.40 | 0.24-0.59 | -1.00 | .317 | 64.00 | 3 | <.001 | 95.31 |
|  | 2000s | 11 | 757 | 0.46 | 0.34-0.59 | -0.56 | .573 | 94.00 | 10 | <.001 | 89.36 |
|  | 2010s | 4 | 116 | 0.36 | 0.16-0.64 | -0.97 | .331 | 16.30 | 3 | .001 | 81.60 |
| Post 5-days up to 1-month | 1980s | 5 | 715 | 0.41 | 0.23-0.62 | -0.84 | .400 | 55.22 | 4 | <.001 | 92.76 |
|  | 1990s | 27 | 4412 | 0.41 | 0.33-0.51 | -1.81 | .070 | 698.34 | 26 | <.001 | 96.28 |
|  | 2000s | 41 | 4077 | 0.42 | 0.35-0.48 | -2.48 | .013 | 528.45 | 40 | <.001 | 92.43 |
|  | 2010s | 15 | 1861 | 0.29 | 0.19-0.41 | -3.37 | <.001 | 228.31 | 14 | <.001 | 93.87 |
| Post 1-month to 4-months | 1980s | 5 | 441 | 0.28 | 0.17-0.41 | -3.13 | .002 | 13.01 | 4 | .011 | 69.25 |
|  | 1990s | 30 | 4767 | 0.26 | 0.22-0.31 | -8.99 | <.001 | 271.12 | 29 | <.001 | 89.30 |
|  | 2000s | 30 | 3944 | 0.26 | 0.21-0.32 | -7.50 | <.001 | 334.77 | 29 | <.001 | 91.34 |
|  | 2010s | 6 | 506 | 0.18 | 0.11-0.29 | -4.91 | <.001 | 21.26 | 5 | .001 | 76.48 |
| Post 4-months to 6-months | 1980s | 3 | 523 | 0.10 | 0.03-0.30 | -3.18 | .001 | 16.51 | 2 | <.001 | 87.89 |
|  | 1990s | 10 | 2202 | 0.20 | 0.15-0.27 | -7.41 | <.001 | 77.30 | 9 | <.001 | 88.36 |
|  | 2000s | 11 | 1118 | 0.19 | 0.11-0.30 | -4.69 | <.001 | 117.99 | 10 | <.001 | 91.52 |
|  | 2010s | 1 | 124 | 0.24 | 0.17-0.32 | -5.48 | <.001 | <0.001 | 0 | 1 | 0.00 |
| Post 6-months to 1-year | 1980s | 1 | 66 | 0.35 | 0.25-0.47 | -2.40 | .016 | <0.001 | 0 | 1 | 0.00 |
|  | 1990s | 4 | 962 | 0.26 | 0.18-0.35 | -4.60 | <.001 | 25.83 | 3 | <.001 | 88.38 |
|  | 2000s | 3 | 1553 | 0.13 | 0.11-0.15 | -25.08 | <.001 | 1.64 | 2 | .441 | 0.00 |
|  | 2010s | 3 | 358 | 0.35 | 0.11-0.71 | -0.79 | .430 | 72.14 | 2 | <.001 | 97.22 |
| Post 1-year to 3-years | 1980s | - | - | - | - | - | - | - | - | - | - |
|  | 1990s | 2 | 123 | 0.48 | 0.39-0.57 | -0.45 | .656 | 0.51 | 1 | .476 | 0.00 |
|  | 2000s | 1 | 32 | 0.31 | 0.18-0.49 | -2.09 | .036 | <0.001 | 0 | 1 | 0.00 |
|  | 2010s | 1 | 48 | 0.27 | 0.16-0.41 | -3.06 | .002 | <0.001 | 0 | 1 | 0.00 |
| Post 3-years to 5-years | 1980s | 1 | 172 | 0.42 | 0.35-0.50 | -2.09 | .037 | <0.001 | 0 | 1 | 0.00 |
|  | 1990s | 2 | 371 | 0.42 | 0.37-0.48 | -2.89 | .004 | <0.001 | 1 | .859 | 0.00 |
|  | 2000s | 2 | 106 | 0.14 | 0.02-0.62 | -1.55 | .120 | 4.92 | 1 | .027 | 79.68 |
|  | 2010s | - | - | - | - | - | - | - | - | - | - |
| Post 5-years | 1980s | - | - | - | - | - | - | - | - | - | - |
|  | 1990s | 1 | 96 | 0.06 | 0.03-0.13 | -6.43 | <.001 | 0.00 | 0 | 1 | 0.00 |
|  | 2000s | 1 | 189 | 0.33 | 0.26-0.40 | -4.63 | <.001 | 0.00 | 0 | 1 | 0.00 |
|  | 2010s | - | - | - | - | - | - | - | - | - | - |

Supplementary Reference List

1 Ahlgren E, Lundqvist A, Nordlund A, et al. Neurocognitive impairment and driving performance after coronary artery bypass surgery. *Eur J Cardiothorac Surg* 2003;**23**:334-40.

2 Al Tmimi L, Van de Velde M, Meyns B, et al. Serum protein S100 as marker of postoperative delirium after off-pump coronary artery bypass surgery: secondary analysis of two prospective randomized controlled trials. *Clin Chem Lab Med* 2016;**54**:1671-80.

3 Alex J, Laden G, Cale A, et al. Pretreatment with hyperbaric oxygen and its effect on neuropsychometric dysfunction and systemic inflammatory response after cardiopulmonary bypass: a prospective randomized double-blind trial. *J Thorac Cardiovasc Surg* 2005;**130**:1623-30.

4 Anastasiadis K, Argiriadou H, Kosmidis M, et al. Neurocognitive outcome after coronary artery bypass surgery using minimal versus conventional extracorporeal circulation: a randomised controlled pilot study. *Heart* 2011;**97**:1082-8.

5 Andrew M, Baker R, Bennetts J, et al. A comparison of neuropsychologic deficits after extracardiac and intracardiac surgery. *J Cardiothorac Vasc Anesth* 2001;**15**:9-14.

6 Arrowsmith J, Harrison M, Newman S, et al. Neuroprotection of the brain during cardiopulmonary bypass: a randomized trial of remacemide during coronary artery bypass in 171 patients. *Stroke* 1998;**29**:2357-62.

7 Aykut K, Albayrak G, Guzeloglu M, et al. Pulsatile versus non-pulsatile flow to reduce cognitive decline after coronary artery bypass surgery: A randomized prospective clinical trial. *J Cardiovasc Dis Res* 2013;**4**:127-9.

8 Baba T, Goto T, Maekawa K, et al. Early neuropsychological dysfunction in elderly high-risk patients after on-pump and off-pump coronary bypass surgery. *J Anesth* 2007;**21**:452-8.

9 Bassano C, Bovio E, Uva F, et al. Partially anaortic clampless off-pump coronary artery bypass prevents neurologic injury compared to on-pump coronary surgery: a propensity score-matched study on 286 patients. *Heart Vessels* 2016;**31**:1412-7.

10 Bi Q, Li JY, Li XQ, et al. Impact of Intracranial Artery Disease and Prior Cerebral Infarction on Central Nervous System Complications After Off-Pump Coronary Artery Bypass Grafting. *Neurophysiology* 2014;**46**:501-6.

11 Bily B, Artemiou P, Sabol F, et al. The role of dexmedetomidine in the prevention of postoperative delirium in cardiac surgery patients. . *Cardiology Letters* 2015;**24**:435-44.

12 Bonacchi M, Prifti E, Maiani M, et al. Does off-pump coronary revascularization reduce the release of the cerebral markers, S-100beta and NSE? *Heart Lung Circ* 2006;**15**:314-9.

13 Boodhwani M, Rubens FD, Wozny D, et al. Predictors of early neurocognitive deficits in low-risk patients undergoing on-pump coronary artery bypass surgery. *Circulation* 2006;**114**:461-6.

14 Borger MA, Peniston CM, Weisel RD, et al. Neuropsychologic impairment after coronary bypass surgery: effect of gaseous microemboli during perfusionist interventions. *J Thorac Cardiovasc Surg* 2001;**121**:743-9.

15 Borger MA, Rao V. Temperature management during cardiopulmonary bypass: Effect of rewarming rate on cognitive dysfunction. *Semin Cardiothorac Vasc Anesth* 2002;**6**:17-20.

16 Braekken SK, Reinvang I, Russell D, et al. Association between intraoperative cerebral microembolic signals and postoperative neuropsychological deficit: comparison between patients with cardiac valve replacement and patients with coronary artery bypass grafting. *J Neurol Neurosurg Psychiatry* 1998;**65**:573-6.

17 Breu A, Stransky M, Metterlein T, et al. Subsyndromal delirium after cardiac surgery. *Scand Cardiovasc J* 2015;**49**:207-12.

18 Breuer AC, Furlan AJ, Hanson MR, et al. Central nervous system complications of coronary artery bypass graft surgery: prospective analysis of 421 patients. *Stroke* 1983;**14**:682-7.

19 Brown CH, Laflam A, Max L, et al. The Impact of Delirium After Cardiac Surgical Procedures on Postoperative Resource Use. *Ann Thorac Surg* 2016;**101**:1663-9.

20 Bruce KM, Yelland GW, Smith JA, et al. Recovery of cognitive function after coronary artery bypass graft operations. *Ann Thorac Surg* 2013;**95**:1306-13.

21 Bucerius J, Gummert J, Borger M, et al. Predictors of delirium after cardiac surgery delirium: Effect of beating-heart (off-pump) surgery. *J Thorac Cardiovasc Surg* 2004;**127**:57-64.

22 Bucerius J, Gummert JF, Walther T, et al. Diabetes in patients undergoing coronary artery bypass grafting. Impact on perioperative outcome. *Z Kardiol* 2005;**94**:575-82.

23 Butterworth J, Legault C, Stump DA, et al. A randomized, blinded trial of the antioxidant pegorgotein: No reduction in neuropsychological deficits, inotropic drug support, or myocardial ischemia after coronary artery bypass surgery. *J Cardiothorac Vasc Anesth* 1999;**13**:690-4.

24 Butterworth J, Wagenknecht LE, Legault C, et al. Attempted control of hyperglycemia during cardiopulmonary bypass fails to improve neurologic or neurobehavioral outcomes in patients without diabetes mellitus undergoing coronary artery bypass grafting. *J Thorac Cardiovasc Surg* 2005;**130**:1319-25.

25 Buziashvili I, Ambat'ello S, Aleksakhina A, et al. Influence of cardiopulmonary bypass on cognitive functions in patients with ischemic heart disease. *Zh Nevrol Psikhiatr Im S S Korsakova* 2005;**105**:30-5.

26 Chakravarthy M, Manjunath V, Jawali V, et al. Neurocognitive behaviour changes in patients undergoing off pump coronary artery bypass surgery- a prospective observational study. *J Anaesthesiol Clin Pharmacol* 2008;**24**:49-52.

27 Chernov VI, Efimova NY, Efimova IY, et al. Short-term and long-term cognitive function and cerebral perfusion in off-pump and on-pump coronary artery bypass patients. *Eur J Cardiothorac Surg* 2006;**29**:74-81.

28 Christiansen C, Berg R, Plovsing R, et al. Dynamic Cerebral Autoregulation after Cardiopulmonary Bypass. *J Thorac cardiovasc Surg* 2016;**64**:569-74.

29 Colak Z, Borojevic M, Bogovic A, et al. Influence of intraoperative cerebral oximetry monitoring on neurocognitive function after coronary artery bypass surgery: a randomized, prospective study. *Eur J Cardiothorac Surg* 2015;**47**:447-54.

30 Cook DJ, Huston J, Trenerry MR, et al. Postcardiac surgical cognitive impairment in the aged using diffusion-weighted magnetic resonance imaging. *Ann Thorac Surg* 2007;**83**:1389-95.

31 Cumurcu BE, Karlidag R, Unal S, et al. Plasma Iron, Copper, Zinc Levels in Patients Experiencing Delirium Following Coronary Artery Bypass Grafting. *Neurol Psychiat Br* 2008;**15**:167-74.

32 Dabrowski W, Rzecki Z, Czajkowski M, et al. Plasma matrix metalloproteinase 9 correlates with disorders of brain magnesium homeostasis in patients undergoing coronary artery bypass surgery. *Magnes Res* 2010;**23**:169-79.

33 de Tournay-Jette E, Dupuis G, Bherer L, et al. The relationship between cerebral oxygen saturation changes and postoperative cognitive dysfunction in elderly patients after coronary artery bypass graft surgery. *J Cardiothorac Vasc Anesth* 2011;**25**:95-104.

34 de Baar M, Diephuis JC, Moons KG, et al. The effect of zero-balanced ultrafiltration during cardiopulmonary bypass on S100b release and cognitive function. *Perfusion* 2003;**18**:9-14.

35 de Tournay-Jetté E, Dupuis G, Denault A, et al. The benefits of cognitive training after a coronary artery bypass graft surgery. *J Behav Med* 2012;**35**:557-68.

36 Dieleman J, Sauer AM, Klijn C, et al. Presence of coronary collaterals is associated with a decreased incidence of cognitive decline after coronary artery bypass surgery. *Eur J Cardiothorac Surg* 2009;**35**:48-53.

37 Djaiani G, Fedorko L, Borger MA, et al. Continuous-flow cell saver reduces cognitive decline in elderly patients after coronary bypass surgery. *Circulation* 2007;**116**:1888-95.

38 Djaiani G, Ali M, Borger MA, et al. Epiaortic scanning modifies planned intraoperative surgical management but not cerebral embolic load during coronary artery bypass surgery. *Anesth Analg* 2008;**106**:1611-8.

39 Djaiani G, Katznelson R, Fedorko L, et al. Early benefit of preserved cognitive function is not sustained at one-year after cardiac surgery: a longitudinal follow-up of the randomized controlled trial. *Can J Anaesth* 2012;**59**:449-55.

40 Dong S, Li CL, Liang WD, et al. Postoperative plasma copeptin levels independently predict delirium and cognitive dysfunction after coronary artery bypass graft surgery. *Peptides* 2014;**59**:70-4.

41 Dowd NP, Karski JM, Cheng DC, et al. Fast-track cardiac anaesthesia in the elderly: effect of two different anaesthetic techniques on mental recovery. *Br J Anaesth* 2001;**86**:68-76.

42 Dumas A, Dupuis GH, Searle N, et al. Early versus late extubation after coronary artery bypass grafting: effects on cognitive function. *J Cardiothorac Vasc Anesth* 1999;**13**:130-5.

43 Ebert AD, Walzer TA, Huth C, et al. Early neurobehavioral disorders after cardiac surgery: a comparative analysis of coronary artery bypass graft surgery and valve replacement. *J Cardiothorac Vasc Anesth* 2001;**15**:15-9.

44 Eriksson M, Samuelsson E, Gustafson Y, et al. Delirium after coronary bypass surgery evaluated by the organic brain syndrome protocol. *Scand Cardiovasc J* 2002;**36**:250-5.

45 Eryomina OV, Petrova MM, Prokopenko SV, et al. The effectiveness of the correction of cognitive impairment using computer-based stimulation programs for patients with coronary heart disease after coronary bypass surgery. *J Neurol Sci* 2015;**358**:188-92.

46 Evered LA, Silbert BS, Scott DA, et al. Plasma amyloid beta42 and amyloid beta40 levels are associated with early cognitive dysfunction after cardiac surgery. *Ann Thorac Surg* 2009;**88**:1426-32.

47 Evered LA, Silbert BS, Scott DA. Postoperative cognitive dysfunction and aortic atheroma. *Ann Thorac Surg* 2010;**89**:1091-7.

48 Evered L, Scott DA, Silbert B, et al. Postoperative Cognitive Dysfunction Is Independent of Type of Surgery and Anesthetic. *Anesth Analg* 2011;**112**:1179-85.

49 Evered L, Silbert B, Scott D, et al. Prevalence of Dementia 7.5 Years after Coronary Artery Bypass Graft Surgery. *Anesthesiology* 2016;**125**:62-71.

50 Farhoudi M, Mehrvar K, Afrasiabi A, et al. Neurocognitive impairment after off-pump and on-pump coronary artery bypass graft surgery - an Iranian experience. *Neuropsychiatr Dis Treat* 2010;**6**:775-8.

51 Forrest CM, Mackay GM, Oxford L, et al. Kynurenine metabolism predicts cognitive function in patients following cardiac bypass and thoracic surgery. *J Neurochem* 2011;**119**:136-52.

52 Gasparovic H, Borojevic M, Malojcic B, et al. Single aortic clamping in coronary artery bypass surgery reduces cerebral embolism and improves neurocognitive outcomes. *Vasc Med* 2013;**18**:275-81.

53 Ghafari R, Baradari AG, Firouzian A, et al. Cognitive deficit in first-time coronary artery bypass graft patients: a randomized clinical trial of lidocaine versus procaine hydrochloride. *Perfusion* 2012;**27**:320-5.

54 Goto T, Baba T, Yoshitake A, et al. Craniocervical and aortic atherosclerosis as neurologic risk factors in coronary surgery. *Ann Thorac Surg* 2000;**69**:834-40.

55 Goto T, Baba T, Honma K, et al. Magnetic resonance imaging findings and postoperative neurologic dysfunction in elderly patients undergoing coronary artery bypass grafting. *Ann Thorac Surg* 2001;**72**:137-42.

56 Goto T, Baba T, Matsuyama K, et al. Aortic atherosclerosis and postoperative neurological dysfunction in elderly coronary surgical patients. *Ann Thorac Surg* 2003;**75**:1912-8.

57 Grigore AM, Grocott HP, Mathew JP, et al. The rewarming rate and increased peak temperature alter neurocognitive outcome after cardiac surgery. *Anesth Analg* 2002;**94**:4-10.

58 Grocott HP, Mackensen GB, Grigore AM, et al. Postoperative hyperthermia is associated with cognitive dysfunction after coronary artery bypass graft surgery. *Stroke* 2002;**33**:537-41.

59 Gunaydin B, Babacan A. Cerebral hypoperfusion after cardiac surgery and anesthetic strategies: a comparative study with high dose fentanyl and barbiturate anesthesia. *Ann Thorac Cardiovasc Surg* 1998;**4**:12-7.

60 Habib S, Khan A, Afridi MI, et al. Frequency and predictors of cognitive decline in patients undergoing coronary artery bypass graft surgery. *J Coll Physicians Surg Pak* 2014;**24**:543-8.

61 Haljan G, Maitland A, Buchan A, et al. The erythropoietin neuroprotective effect: assessment in CABG surgery (TENPEAKS): a randomized, double-blind, placebo controlled, proof-of-concept clinical trial. *Stroke* 2009;**40**:2769-75.

62 Hall RA, Fordyce DJ, Lee ME, et al. Brain SPECT imaging and neuropsychological testing in coronary artery bypass patients: single photon emission computed tomography. *Ann Thorac Surg* 1999;**68**:2082-8.

63 Hall MW, Hopkins RO, Long JW, et al. Hypothermia-induced platelet aggregation and cognitive decline in coronary artery bypass surgery: a pilot study. *Perfusion* 2005;**20**:157-67.

64 Hammon JW, Stump DA, Butterworth JF, et al. Single crossclamp improves 6-month cognitive outcome in high-risk coronary bypass patients: the effect of reduced aortic manipulation. *J Thorac Cardiovasc Surg* 2006;**131**:114-21.

65 Harmon DC, Ghori KG, Eustace NP, et al. Aprotinin decreases the incidence of cognitive deficit following CABG and cardiopulmonary bypass: a pilot randomized controlled study. *Can J Anaesth* 2004;**51**:1002-9.

66 Harmon D, Eustace N, Ghori K, et al. Plasma concentrations of nitric oxide products and cognitive dysfunction following coronary artery bypass surgery. *Eur J Anaesthesiol* 2005;**22**:269-76.

67 Harrison MJ, Schneidau A, Ho R, et al. Cerebrovascular disease and functional outcome after coronary artery bypass surgery. *Stroke* 1989;**20**:235-7.

68 Hernandez F, Jr., Brown JR, Likosky DS, et al. Neurocognitive outcomes of off-pump versus on-pump coronary artery bypass: a prospective randomized controlled trial. *Ann Thorac Surg* 2007;**84**:1897-903.

69 Herrmann M, Ebert AD, Galazky I, et al. Neurobehavioral outcome prediction after cardiac surgery: role of neurobiochemical markers of damage to neuronal and glial brain tissue. *Stroke* 2000;**31**:645-50.

70 Heyer EJ, Adams DC, Delphin E, et al. Cerebral dysfunction after coronary artery bypass grafting done with mild or moderate hypothermia. *J Thorac Cardiovasc Surg* 1997;**114**:270-7.

71 Ho P, Arciniegas D, Grigsby J, et al. Predictors of cognitive decline following coronary artery bypass graft surgery. *Ann Thorac Surg* 2004;**77**:597-603.

72 Hudetz JA, Iqbal Z, Gandhi SD, et al. Postoperative Delirium and Short-term Cognitive Dysfunction Occur More Frequently in Patients Undergoing Valve Surgery With or Without Coronary Artery Bypass Graft Surgery Compared With Coronary Artery Bypass Graft Surgery Alone: Results of a Pilot Study. *J Cardiothorac Vasc Anesth* 2011;**25**:811-6.

73 Humphreys JM, Denson LA, Baker RA, et al. The importance of depression and alcohol use in coronary artery bypass graft surgery patients: risk factors for delirium and poorer quality of life. *J Geriatr Cardiol* 2016;**13**:51-7.

74 Ito A, Goto T, Maekawa K, et al. Postoperative neurological complications and risk factors for pre-existing silent brain infarction in elderly patients undergoing coronary artery bypass grafting. *J Anesth* 2012;**26**:405-11.

75 Jensen BO, Hughes P, Rasmussen LS, et al. Cognitive outcomes in elderly high-risk patients after off-pump versus conventional coronary artery bypass grafting: a randomized trial. *Circulation* 2006;**113**:2790-5.

76 Jensen B, Rasmussen L, Steinbrüchel D. Cognitive outcomes in elderly high-risk patients 1 year after off-pump versus on-pump coronary artery bypass grafting. A randomized trial. *Eur J Cardiothorac Surg* 2008;**34**:1016-21.

77 Jonsson H, Johnsson P, Backstrom M, et al. Controversial significance of early S100B levels after cardiac surgery. *BMC Neurol* 2004;**4**:24.

78 Joung KW, Rhim JH, Chin JH, et al. Effect of remote ischemic preconditioning on cognitive function after off-pump coronary artery bypass graft: a pilot study. *Korean J Anesthesiol* 2013;**65**:418-24.

79 Kadoi Y, Saito S, Goto F, et al. Decrease in jugular venous oxygen saturation during normothermic cardiopulmonary bypass predicts short-term postoperative neurologic dysfunction in elderly patients. *J Am Coll Cardiol* 2001;**38**:1450-5.

80 Kadoi Y, Saito S, Goto F, et al. Slow rewarming has no effects on the decrease in jugular venous oxygen hemoglobin saturation and long-term cognitive outcome in diabetic patients. *Anesth Analg* 2002;**94**:1395-401.

81 Kadoi Y, Saito S, Kunimoto F, et al. Comparative effects of propofol versus fentanyl on cerebral oxygenation state during normothermic cardiopulmonary bypass and postoperative cognitive dysfunction. *Ann Thorac Surg* 2003;**75**:840-6.

82 Kadoi Y, Saito S, Fujita N, et al. Risk factors for cognitive dysfunction after coronary artery bypass graft surgery in patients with type 2 diabetes. *J Thorac Cardiovasc Surg* 2005;**129**:576-83.

83 Kadoi Y, Goto F. Sevoflurane anesthesia did not affect postoperative cognitive dysfunction in patients undergoing coronary artery bypass graft surgery. *J Anesth* 2007;**21**:330-5.

84 Kadoi Y, Kawauchi C, Ide M, et al. Preoperative depression is a risk factor for postoperative short-term and long-term cognitive dysfunction in patients with diabetes mellitus. *J Anesth* 2011;**25**:10-7.

85 Kadoi Y, Kawauchi C, Kuroda M, et al. Association between cerebrovascular carbon dioxide reactivity and postoperative short-term and long-term cognitive dysfunction in patients with diabetes mellitus. *J Anesth* 2011;**25**:641-7.

86 Kadoi Y, Saito S, Fujita N, et al. Effects of balloon-induced pulsatile perfusion on postoperative short- and long-term cognitive dysfunction in diabetic patients with impaired cerebrovascular carbon dioxide reactivity. *J Cardiothorac Vasc Anesth* 2013;**27**:238-44.

87 Kara I, Erkin A, Sacli H, et al. The Effects of Near-Infrared Spectroscopy on the Neurocognitive Functions in the Patients Undergoing Coronary Artery Bypass Grafting with Asymptomatic Carotid Artery Disease: A Randomized Prospective Study. *Ann Thorac Cardiovasc Surg* 2015;**21**:544-50.

88 Kaukinen L, Porkkala H, Kaukinen S, et al. Release of brain-specific creatine kinase and neuron-specific enolase into cerebrospinal fluid after hypothermic and normothermic cardiopulmonary bypass in coronary artery surgery. *Acta Anaesthesiol Scand* 2000;**44**:361-8.

89 Kazmierski J, Banys A, Latek J, et al. Mild Cognitive Impairment with Associated Inflammatory and Cortisol Alterations as Independent Risk Factor for Postoperative Delirium. *Dement Geriatr Cogn* 2014;**38**:65-78.

90 Kazmierski J, Sieruta M, Banys A, et al. The assessment of the T102C polymorphism of the 5HT2a receptor gene, 3723G/A polymorphism of the NMDA receptor 3A subunit gene (GRIN3A) and 421C/A polymorphism of the NMDA receptor 2B subunit gene (GRIN2B) among cardiac surgery patients with and without delirium. *Gen Hosp Psychiatry* 2014;**36**:753-6.

91 Keizer AMA, Hijman R, Kalkman CJ, et al. The incidence of cognitive decline after (not) undergoing coronary artery bypass grafting: The impact of a controlled definition. *Acta Anaesthesiol Scand* 2005;**49**:1232-5.

92 Kempfert J, Opfermann UT, Richter M, et al. Twelve-month patency with the PAS-port proximal connector device: a single center prospective randomized trial. *Ann Thorac Surg* 2008;**85**:1579-84.

93 Khan I, Khan AH, Gull S, et al. Incidence and predictors of Delirium in postoperative coronary artery bypass surgery patients in Pakistani population. *Pakistan Journal of Medical and Health Sciences* 2014;**8**:92-7.

94 Khatri P, Babyak M, Clancy C, et al. Perception of cognitive function in older adults following coronary artery bypass surgery. *Health Psychol* 1999;**18**:301-6.

95 Knipp SC, Matatko N, Wilhelm H, et al. Cognitive outcomes three years after coronary artery bypass surgery: relation to diffusion-weighted magnetic resonance imaging. *Ann Thorac Surg* 2008;**85**:872-9.

96 Kok WF, van Harten AE, Koene BMJA, et al. A pilot study of cerebral tissue oxygenation and postoperative cognitive dysfunction among patients undergoing coronary artery bypass grafting randomised to surgery with or without cardiopulmonary bypass*. *Anaesthesia* 2014;**69**:613-22.

97 Kok WF, Koerts J, Tucha O, et al. Neuronal damage biomarkers in the identification of patients at risk of long-term postoperative cognitive dysfunction after cardiac surgery. *Anaesthesia* 2017;**72**:359-69.

98 Kozora E, Kongs S, Collins JF, et al. Cognitive outcomes after on- versus off-pump coronary artery bypass surgery. *Ann Thorac Surg* 2010;**90**:1134-41.

99 Krzych LJ, Wybraniec MT, Krupka-Matuszczyk I, et al. Detailed Insight Into the Impact of Postoperative Neuropsychiatric Complications on Mortality in a Cohort of Cardiac Surgery Subjects: A 23,000-Patient-Year Analysis. *J Cardiothorac Vasc Anesth* 2014;**28**:448-57.

100 Lahariya S, Grover S, Bagga S, et al. Delirium in patients admitted to a cardiac intensive care unit with cardiac emergencies in a developing country: incidence, prevalence, risk factor and outcome. *Gen Hosp Psychiatry* 2014;**36**:156-64.

101 Leacche M, Carrier M, Bouchard D, et al. Improving neurologic outcome in off-pump surgery: The "no touch" technique. *Heart Surg Forum* 2003;**6**:169-75.

102 Lee JD, Lee SJ, Tsushima WT, et al. Benefits of off-pump bypass on neurologic and clinical morbidity: a prospective randomized trial. *Ann Thorac Surg* 2003;**76**:18-26.

103 Lee TA, Wolozin B, Weiss KB, et al. Assessment of the emergence of Alzheimer's disease following coronary artery bypass graft surgery or percutaneous transluminal coronary angioplasty. *J Alzheimers Dis* 2005;**7**:319-24.

104 Lelis RG, Krieger JE, Pereira AC, et al. Apolipoprotein E4 genotype increases the risk of postoperative cognitive dysfunction in patients undergoing coronary artery bypass graft surgery. *J Cardiovasc Surg* 2006;**47**:451-6.

105 Lewis MS, Maruff P, Silbert BS, et al. Detection of postoperative cognitive decline after coronary artery bypass graft surgery is affected by the number of neuropsychological tests in the assessment battery. *Ann Thorac Surg* 2006;**81**:2097-104.

106 Lewis MS, Maruff P, Silbert BS, et al. The sensitivity and specificity of three common statistical rules for the classification of post-operative cognitive dysfunction following coronary artery bypass graft surgery. *Acta Anaesthesiol Scand* 2006;**50**:50-7.

107 Lewis MS, Maruff P, Silbert BS, et al. The influence of different error estimates in the detection of postoperative cognitive dysfunction using reliable change indices with correction for practice effects *Arch Clin Neuropsychol* 2007;**22**:249-57.

108 Li HC, Chen YS, Chiu MJ, et al. Delirium, subsyndromal delirium, and cognitive changes in individuals undergoing elective coronary artery bypass graft surgery. *J Cardiovasc Nurs* 2015;**30**:340-5.

109 Liu YH, Wang DX, Li LH, et al. The effects of cardiopulmonary bypass on the number of cerebral microemboli and the incidence of cognitive dysfunction after coronary artery bypass graft surgery. *Anesth Analg* 2009;**109**:1013-22.

110 Loponen P, Luther M, Wistbacka J-O, et al. Postoperative delirium and health related quality of life after coronary artery bypass grafting. *Scand Cardiovasc J* 2008;**42**:337-44.

111 Lund C, Hol PK, Lundblad R, et al. Comparison of cerebral embolization during off-pump and on-pump coronary artery bypass surgery. *Ann Thorac Surg* 2003;**76**:765-70.

112 Lund C, Sundet K, Tennøe B, et al. Cerebral Ischemic Injury and Cognitive Impairment After Off-Pump and On-Pump Coronary Artery Bypass Grafting Surgery. *Ann Thorac Surg* 2005;**80**:2126-31.

113 Maekawa K, Goto T, Baba T, et al. Impaired cognition preceding cardiac surgery is related to cerebral ischemic lesions. *J Anesth* 2011;**25**:330-6.

114 Mahanna EP, Blumenthal JA, White WD, et al. Defining neuropsychological dysfunction after coronary artery bypass grafting. *Ann Thorac Surg* 1996;**61**:1342-7.

115 Mardani D, Bigdelian H. Predictors and clinical outcomes of postoperative delirium after administration of dexamethasone in patients undergoing coronary artery bypass surgery. *Int J Prev Med* 2012;**3**:420-7.

116 Mariscalco G, Cottini M, Zanobini M, et al. Preoperative statin therapy is not associated with a decrease in the incidence of delirium after cardiac operations. *Ann Thorac Surg* 2012;**93**:1439-47.

117 Martens S, Dietrich M, Herzog C, et al. Automatic connector devices for proximal anastomoses do not decrease embolic debris compared with conventional anastomoses in CABG. *Eur J Cardiothorac Surg* 2004;**25**:993-1000.

118 Martin BJ, Buth KJ, Arora RC, et al. Delirium as a predictor of sepsis in post-coronary artery bypass grafting patients: a retrospective cohort study. *Crit Care* 2010;**14**:R171.

119 Martin BJ, Buth KJ, Arora RC, et al. Delirium: a cause for concern beyond the immediate postoperative period. *Ann Thorac Surg* 2012;**93**:1114-20.

120 Mathew JP, Grocott HP, Phillips-Bute B, et al. Lower endotoxin immunity predicts increased cognitive dysfunction in elderly patients after cardiac surgery. *Stroke* 2003;**34**:508-13.

121 Mathew JP, Grocott HP, McCurdy JR, et al. Preoperative statin therapy does not reduce cognitive dysfunction after cardiopulmonary bypass. *J Cardiothorac Vasc Anesth* 2005;**19**:294-9.

122 Mathew JP, Rinder HM, Smith BR, et al. Transcerebral platelet activation after aortic cross-clamp release is linked to neurocognitive decline. *Ann Thorac Surg* 2006;**81**:1644-9.

123 Mathew JP, Podgoreanu MV, Grocott HP, et al. Genetic variants in P-selectin and C-reactive protein influence susceptibility to cognitive decline after cardiac surgery. *J Am Coll Cardiol* 2007;**49**:1934-42.

124 Millar K, Asbury AJ, Murray GD. Pre-existing cognitive impairment as a factor influencing outcome after cardiac surgery. *Br J Anaesth* 2001;**86**:63-7.

125 Miyazaki S, Yoshitani K, Miura N, et al. Risk factors of stroke and delirium after off-pump coronary artery bypass surgery. *Interact Cardiovasc Thorac Surg* 2011;**12**:379-83.

126 Molstrom S, Nielsen TH, Andersen C, et al. Bedside Monitoring of Cerebral Energy State During Cardiac Surgery-A Novel Approach Utilizing Intravenous Microdialysis. *J Cardiothorac Vasc Anesth* 2016:1166-73.

127 Mongero LB, Beck JR, Manspeizer HE, et al. Cardiac surgical patients exposed to heparin-bonded circuits develop less postoperative cerebral dysfunction than patients exposed to non-heparin-bonded circuits. *Perfusion* 2001;**16**:107-11.

128 Mu DL, Wang DX, Li LH, et al. High serum cortisol level is associated with increased risk of delirium after coronary artery bypass graft surgery: a prospective cohort study. *Crit Care* 2010;**14**:R238.

129 Mu DL, Li LH, Wang DX, et al. High Postoperative Serum Cortisol Level Is Associated with Increased Risk of Cognitive Dysfunction Early after Coronary Artery Bypass Graft Surgery: A Prospective Cohort Study. *PLoS One* 2013;**8**:e77637.

130 Müllges W, Babin–Ebell J, Reents W, et al. Cognitive performance after coronary artery bypass grafting: A follow-up study. *Neurology* 2002;**59**:741-3.

131 Murkin JM, Martzke JS, Buchan AM, et al. A randomized study of the influence of perfusion technique and pH management strategy in 316 patients undergoing coronary artery bypass surgery. II. Neurologic and cognitive outcomes. *J Thorac Cardiovasc Surg* 1995;**110**:349-62.

132 Mutch WA, Fransoo RR, Campbell BI, et al. Dementia and depression with ischemic heart disease: a population-based longitudinal study comparing interventional approaches to medical management. *PLoS One* 2011;**6**:e17457.

133 Nathan HJ, Rodriguez R, Wozny D, et al. Neuroprotective effect of mild hypothermia in patients undergoing coronary artery surgery with cardiopulmonary bypass: five-year follow-up of a randomized trial. *J Thorac Cardiovasc Surg* 2007;**133**:1206-11.

134 Newman M, Kirchner J, Phillips-Bute B, et al. Longitudinal Assessment of Neurocognitive Function after Coronary-Artery Bypass Surgery. *N Engl J Med* 2001;**344**:395-402.

135 Nikolić B, Putnik S, Lazovic D, et al. Can we identify risk factors for postoperative delirium in cardiac coronary patients? Our experience. *Heart Surg Forum* 2012:E195-e9.

136 Norkiene I, Ringaitiene D, Misiuriene I, et al. Incidence and precipitating factors of delirium after coronary artery bypass grafting. *Scand Cardiovasc J* 2007;**41**:180-5.

137 Norkiene I, Samalavicius R, Ivaskevicius J, et al. Asymptomatic carotid artery stenosis and cognitive outcomes after coronary artery bypass grafting. *Scand Cardiovasc J* 2011;**45**:169-73.

138 Oh YJ, Kim JY, Shim JK, et al. Diabetes mellitus does not affect jugular bulb oxygen saturation in patients undergoing off-pump coronary artery bypass graft surgery. *Circ J* 2008;**72**:1259-64.

139 Oldham MA, Hawkins KA, Yuh DD, et al. Cognitive and functional status predictors of delirium and delirium severity after coronary artery bypass graft surgery: an interim analysis of the Neuropsychiatric Outcomes After Heart Surgery study. *Int Psychogeriatr* 2015;**27**:1929-38.

140 Omiya H, Yoshitani K, Yamada N, et al. Preoperative brain magnetic resonance imaging and postoperative delirium after off-pump coronary artery bypass grafting: a prospective cohort study. *Can J Anaesth* 2015;**62**:595-602.

141 Osse RJ, Fekkes D, Tulen JH, et al. High preoperative plasma neopterin predicts delirium after cardiac surgery in older adults. *J Am Geriatr Soc* 2012;**60**:661-8.

142 Otomo S, Maekawa K, Goto T, et al. Pre-existing cerebral infarcts as a risk factor for delirium after coronary artery bypass graft surgery. *Interact Cardiovasc Thorac Surg* 2013;**17**:799-804.

143 Ozturk S, Sacar M, Baltalarli A, et al. Effect of the type of cardiopulmonary bypass pump flow on postoperative cognitive function in patients undergoing isolated coronary artery surgery. *Anatol J Cardiol* 2016;**16**:875-80.

144 Palmbergen WA, van Sonderen A, Keyhan-Falsafi AM, et al. Improved perioperative neurological monitoring of coronary artery bypass graft patients reduces the incidence of postoperative delirium: the Haga Brain Care Strategy. *Interact Cardiovasc Thorac Surg* 2012;**15**:671-7.

145 Patel RL, Turtle MR, Chambers DJ, et al. Alpha-stat acid-base regulation during cardiopulmonary bypass improves neuropsychologic outcome in patients undergoing coronary artery bypass grafting. *J Thorac Cardiovasc Surg* 1996;**111**:1267-79.

146 Peterson JC, Pirraglia PA, Wells MT, et al. Attrition in longitudinal randomized controlled trials: home visits make a difference. *Bmc Med Res Methodol* 2012;**12**:178.

147 Phillips-Bute B, Mathew JP, Blumenthal JA, et al. Association of neurocognitive function and quality of life 1 year after coronary artery bypass graft (CABG) surgery. *Psychosom Med* 2006;**68**:369-75.

148 Prakanrattana U, Prapaitrakool S. Efficacy of risperidone for prevention of postoperative delirium in cardiac surgery. *Anaesth Intensive Care* 2007;**35**:714-9.

149 Ramlawi B, Rudolph JL, Mieno S, et al. Serologic markers of brain injury and cognitive function after cardiopulmonary bypass. *Ann Surg* 2006;**244**:593-601.

150 Rasmussen LS, Christiansen M, Hansen PB, et al. Do blood levels of neuron-specific enolase and S-100 protein reflect cognitive dysfunction after coronary artery bypass? *Acta Anaesthesiol Scand* 1999;**43**:495-500.

151 Rasmussen LS, Sperling B, Abildstrom HH, et al. Neuron loss after coronary artery bypass detected by SPECT estimation of benzodiazepine receptors. *Ann Thorac Surg* 2002;**74**:1576-80.

152 Raymond PD, Hinton-Bayre AD, Radel M, et al. Assessment of statistical change criteria used to define significant change in neuropsychological test performance following cardiac surgery. *Eur J Cardiothorac Surg* 2006;**29**:82-8.

153 Reents W, Muellges W, Franke D, et al. Cerebral oxygen saturation assessed by near-infrared spectroscopy during coronary artery bypass grafting and early postoperative cognitive function. *Ann Thorac Surg* 2002;**74**:109-14.

154 Restrepo L, Wityk RJ, Grega MA, et al. Diffusion- and perfusion-weighted magnetic resonance imaging of the brain before and after coronary artery bypass grafting surgery. *Stroke* 2002;**33**:2909-15.

155 Ringaitiene D, Gineityte D, Vicka V, et al. Impact of malnutrition on postoperative delirium development after on pump coronary artery bypass grafting. *J Cardiothorac Surg* 2015;**10**:74.

156 Robson MJA, Alston RP, Deary IJ, et al. Cognition after coronary artery surgery is not related to postoperative jugular bulb oxyhemoglobin desaturation. *Anesth Analg* 2000;**91**:1317-26.

157 Rolfson D, McElhaney J, Jhangri G, et al. Validity of the Confusion Assessment Method in Detecting Postoperative Delirium in the Elderly. *Int Psychogeriatr* 1999;**11**:431-8.

158 Royse AG, Royse CF, Ajani AE, et al. Reduced neuropsychological dysfunction using epiaortic echocardiography and the exclusive Y graft. *Ann Thorac Surg* 2000;**69**:1431-8.

159 Royse CF, Andrews DT, Newman SN, et al. The influence of propofol or desflurane on postoperative cognitive dysfunction in patients undergoing coronary artery bypass surgery. *Anaesthesia* 2011;**66**:455-64.

160 Rudolph JL, Babikian VL, Birjiniuk V, et al. Atherosclerosis is associated with delirium after coronary artery bypass graft surgery. *J Am Geriatr Soc* 2005;**53**:462-6.

161 Rudolph J, Jones R, Grande L, et al. Impaired Executive Function Is Associated with Delirium After Coronary Artery Bypass Graft Surgery. *J Am Geriatr Soc* 2006;**54**:937-41.

162 Rudolph JL, Babikian VL, Treanor P, et al. Microemboli are not associated with delirium after coronary artery bypass graft surgery. *Perfusion* 2009;**24**:409-15.

163 Saczynski J, Marcantonio E, Quach L, et al. Cognitive Trajectories after Postoperative Delirium. *N Engl J Med* 2012;**367**:30-9.

164 Santos S, Velasco T. Clinical features in elderly patients submitted to coronary artery bypass graft (CABG). *Rev Bras Med* 2005;**62**:96-102.

165 Scott DA, Silbert BS, Doyle TJ, et al. Centrifugal versus roller head pumps for cardiopulmonary bypass: effect on early neuropsychologic outcomes after coronary artery surgery. *J Cardiothorac Vasc Anesth* 2002;**16**:715-22.

166 Sellman M, Holm L, Ivert T, et al. A randomized study of neuropsychological function in patients undergoing coronary bypass surgery. *Thorac Cardiovasc Surg* 1993;**41**:349-54.

167 Selnes OA, Grega MA, Bailey MM, et al. Cognition 6 years after surgical or medical therapy for coronary artery disease. *Ann Neurol* 2008;**63**:581-90.

168 Sevuk U, Baysal E, Ay N, et al. Relationship between cobalamin deficiency and delirium in elderly patients undergoing cardiac surgery. *Neuropsychiatr Dis Treat* 2015;**11**:2033-9.

169 Shaw P, Bates D, Cartlidge N, et al. Early intellectual dysfunction following coronary bypass surgery. *Q J Med* 1986;**58**:59-68.

170 Shaw P, Bates D, Cartlidge N, et al. Long-term Intellectual Dysfunction Following Coronary Artery Bypass Graft Surgery: A Six Month Follow-up Study. *QJM-INT J MED* 1987;**62**:259-68.

171 Shioiri A, Kurumaji A, Takeuchi T, et al. A Decrease in the Volume of Gray Matter as a Risk Factor for Postoperative Delirium Revealed by an Atlas-based Method. *Am J Geriatr Psychiatry* 2016;**24**:528-36.

172 Siepe M, Pfeiffer T, Gieringer A, et al. Increased systemic perfusion pressure during cardiopulmonary bypass is associated with less early postoperative cognitive dysfunction and delirium. *Eur J Cardiothorac Surg* 2011;**40**:200-7.

173 Silbert BS, Maruff P, Evered LA, et al. Detection of cognitive decline after coronary surgery: a comparison of computerized and conventional tests. *Br J Anaesth* 2004;**92**:814-20.

174 Silva F, Schmidt A, Valentin L, et al. S100B protein and neuron-specific enolase as predictors of cognitive dysfunction after coronary artery bypass graft surgery: A prospective observational study. *Eur J Anaesthesiol* 2016;**33**:681-9.

175 Sirvinskas E, Usas E, Mankute A, et al. Effects of intraoperative external head cooling on short-term cognitive function in patients after coronary artery bypass graft surgery. *Perfusion* 2014;**29**:124-9.

176 Slater JP, Guarino T, Stack J, et al. Cerebral oxygen desaturation predicts cognitive decline and longer hospital stay after cardiac surgery. *Ann Thorac Surg* 2009;**87**:36-45.

177 Smith P, Treasure T, Newman S, et al. Cerebral consequences of cardiopulmonary bypass. *Lancet* 1986;**1**:823-5.

178 Smith PL. The cerebral complications of coronary artery bypass surgery. *Ann R Coll Surg Engl* 1988;**70**:212-6.

179 Soehle M, Dittmann A, Ellerkmann RK, et al. Intraoperative burst suppression is associated with postoperative delirium following cardiac surgery: a prospective, observational study. *Bmc Anesthesiol* 2015;**15**:61.

180 Song Z, Fu P, Chen M, et al. Association of CT perfusion and postoperative cognitive dysfunction after off-pump coronary artery bypass grafting. *Neurol Res* 2016;**38**:533-7.

181 Stanley TO, Mackensen GB, Grocott HP, et al. The impact of postoperative atrial fibrillation on neurocognitive outcome after coronary artery bypass graft surgery. *Anesth Analg* 2002;**94**:290-5.

182 Stroobant N, Van Nooten G, Belleghem Y, et al. Short-term and long-term neurocognitive outcome in on-pump versus off-pump CABG. *Eur J Cardiothorac Surg* 2002;**22**:559-64.

183 Stroobant N, Van Nooten G, Van Belleghem Y, et al. Relation between neurocognitive impairment, embolic load, and cerebrovascular reactivity following on- and off-pump coronary artery bypass grafting. *Chest* 2005;**127**:1967-76.

184 Stroobant N, van Nooten G, De Bacquer D, et al. Neuropsychological functioning 3-5 years after coronary artery bypass grafting: does the pump make a difference? *Eur J Cardiothorac Surg* 2008;**34**:396-401.

185 Suksompong S, Prakanratrana U, Chumpathong S, et al. Neuropsychological alterations after coronary artery bypass graft surgery. *Journal of the Medical Association of Thailand = Chotmaihet thangphaet* 2002;**85 Suppl 3**:S910-S6.

186 Svenmarker S, Engstrom K, Karlsson T, et al. Influence of pericardial suction blood retransfusion on memory function and release of protein S100B. *Perfusion* 2004;**19**:337-43.

187 Swaminathan M, McCreath BJ, Phillips-Bute BG, et al. Serum creatinine patterns in coronary bypass surgery patients with and without postoperative cognitive dysfunction. *Anesth Analg* 2002;**95**:1-8.

188 Sylivris S, Levi C, Matalanis G, et al. Pattern and significance of cerebral microemboli during coronary artery bypass grafting. *Ann Thorac Surg* 1998;**66**:1674-8.

189 Szwed K, Pawliszak W, Anisimowicz L, et al. Short-term outcome of attention and executive functions from aorta no-touch and traditional off-pump coronary artery bypass surgery. *World J Biol Psychiatry* 2014;**15**:397-403.

190 Tabatabaie O, Matin N, Heidari A, et al. Spinal anesthesia reduces postoperative delirium in opium dependent patients undergoing coronary artery bypass grafting. *Acta Anaesthesiol Belg* 2015;**66**:49-54.

191 Tagarakis GI, Tsolaki-Tagaraki F, Tsolaki M, et al. The role of apolipoprotein E in cognitive decline and delirium after bypass heart operations. *Am J Alzheimers Dis Other Demen* 2007;**22**:223-8.

192 Taggart DP, Browne SM, Wade DT, et al. Neuroprotection during cardiac surgery: a randomised trial of a platelet activating factor antagonist. *Heart* 2003;**89**:897-900.

193 Tan MC, Felde A, Kuskowski M, et al. Incidence and predictors of post-cardiotomy delirium. *Am J Geriatr Psychiatry* 2008;**16**:575-83.

194 Thornton EW, Groom C, Fabri BM, et al. Quality of life outcomes after coronary artery bypass graft surgery: relationship to neuropsychologic deficit. *J Thorac Cardiovasc Surg* 2005;**130**:1022-7.

195 Toeg HD, Nathan H, Rubens F, et al. Clinical impact of neurocognitive deficits after cardiac surgery. *J Thorac Cardiovasc Surg* 2013;**145**:1545-9.

196 Toner I, Peden CJ, Hamid SK, et al. Magnetic resonance imaging and neuropsychological changes after coronary artery bypass graft surgery: preliminary findings. *J Neurosurg Anesthesiol* 1994;**6**:163-9.

197 Toner I, Taylor KM, Lockwood G, et al. EEG changes during cardiopulmonary bypass surgery and postoperative neuropsychological deficit: the effect of bubble and membrane oxygenators. *Eur J Cardiothorac Surg* 1997;**11**:312-9.

198 Toner I, Taylor KM, Newman S, et al. Cerebral functional changes following cardiac surgery: Neuropsychological and EEG assessment. *Eur J Cardiothorac Surg* 1998;**13**:13-20.

199 Trubnikova O, Mamontova A, Syrova I, et al. Does preoperative mild cognitive impairment predict postoperative cognitive dysfunction after on-pump coronary bypass surgery? *J Alzheimers Dis* 2014;**42**:S45-S51.

200 Tse L, Schwarz SKW, Bowering JB, et al. Incidence of and Risk Factors for Delirium After Cardiac Surgery at a Quaternary Care Center: A Retrospective Cohort Study. *J Cardiothorac Vasc Anesth* 2015;**29**:1472-9.

201 Tully PJ, Baker RA, Eld HRW, et al. Depression, anxiety disorders and Type D personality as risk factors for delirium after cardiac surgery. *Aust Nz J Psychiat* 2010;**44**:1005-11.

202 Vanninen R, Aikia M, Kononen M, et al. Subclinical cerebral complications after coronary artery bypass grafting: prospective analysis with magnetic resonance imaging, quantitative electroencephalography, and neuropsychological assessment. *Arch Neurol* 1998;**55**:618-27.

203 van Dijk D, Jansen E, Hijman R, et al. Cognitive outcome after off-pump and on-pump coronary artery bypass graft surgery: A randomized trial. *JAMA* 2002;**287**:1405-12.

204 van Dijk D, Moons K, Keizer A, et al. Association between early and three month cognitive outcome after off-pump and on-pump coronary bypass surgery. *Heart* 2004;**90**:431-4.

205 van Dijk D, Spoor M, Hijman R, et al. Cognitive and cardiac outcomes 5 years after off-pump vs on-pump coronary artery bypass graft surgery. *JAMA* 2007;**297**:701-8.

206 Vedin J, Nyman H, Ericsson A, et al. Cognitive function after on or off pump coronary artery bypass grafting. *Eur J Cardiothorac Surg* 2006;**30**:305-10.

207 Walzer T, Herrmann M, Wallesch CW. Neuropsychological disorders after coronary bypass surgery. *J Neurol Neurosurg Psychiatry* 1997;**62**:644-8.

208 Wang D, Wu X, Li J, et al. The effect of lidocaine on early postoperative cognitive dysfunction after coronary artery bypass surgery. *Anesth Analg* 2002;**95**:1134-41.

209 Whitaker DC, Newman SP, Stygall J, et al. The effect of leucocyte-depleting arterial line filters on cerebral microemboli and neuropsychological outcome following coronary artery bypass surgery. *Eur J Cardiothorac Surg* 2004;**25**:267-74.

210 Yilmaz S, Aksoy E, Diken AI, et al. Dopamine Administration is a Risk Factor for Delirium in Patients Undergoing Coronary Artery Bypass Surgery. *Heart Lung Circ* 2016;**25**:493-8.

211 Yoda M, Nonoyama M, Shimakura T. Cerebral perfusion during off-pump coronary artery bypass grafting. *Surg Today* 2004;**34**:501-5.

212 Yoon BW, Bae HJ, Kang DW, et al. Intracranial cerebral artery disease as a risk factor for central nervous system complications of coronary artery bypass graft surgery. *Stroke* 2001;**32**:94-9.

213 Zamvar V, Williams D, Hall J, et al. Assessment of neurocognitive impairment after off-pump and on-pump techniques for coronary artery bypass graft surgery: prospective randomised controlled trial. *BMJ* 2002;**325**:1268.

214 Zhang WY, Wu WL, Gu JJ, et al. Risk factors for postoperative delirium in patients after coronary artery bypass grafting: A prospective cohort study. *J Crit Care* 2015;**30**:606-12.

215 Zimpfer D, Czerny M, Vogt F, et al. Neurocognitive deficit following coronary artery bypass grafting: a prospective study of surgical patients and nonsurgical controls. *Ann Thorac Surg* 2004;**78**:513-9.
